# Mechanistic Insights Into Oxidative Response of Heat Shock Factor 1 Condensates

**DOI:** 10.1021/jacsau.4c00578

**Published:** 2025-01-30

**Authors:** Soichiro Kawagoe, Motonori Matsusaki, Takuya Mabuchi, Yuto Ogasawara, Kazunori Watanabe, Koichiro Ishimori, Tomohide Saio

**Affiliations:** † Institute of Advanced Medical Sciences, Tokushima University, Tokushima 770-8503, Japan; ‡ Frontier Research Institute for Interdisciplinary Sciences, Tohoku University, 2-1-1 Katahira, Aoba-ku, Sendai, Miyagi 980-8577, Japan; § Institute of Fluid Science, Tohoku University, 2-1-1 Katahira, Aoba-ku, Sendai, Miyagi 980-8577, Japan; ∥ Department of Interdisciplinary Science and Engineering in Health Systems, Okayama University, 3-1-1 Tsushimanaka, Okayama 700-8530, Japan; ⊥ Department of Chemistry, Faculty of Science, Hokkaido University, Sapporo, Hokkaido 060-0810, Japan; # Fujii Memorial Institute of Medical Sciences, Institute of Advanced Medical Sciences, Tokushima University, Tokushima 770-8503, Japan

**Keywords:** heat shock factor 1, oxidative hyper-oligomerization, biological phase transition, stress response, biophysics

## Abstract

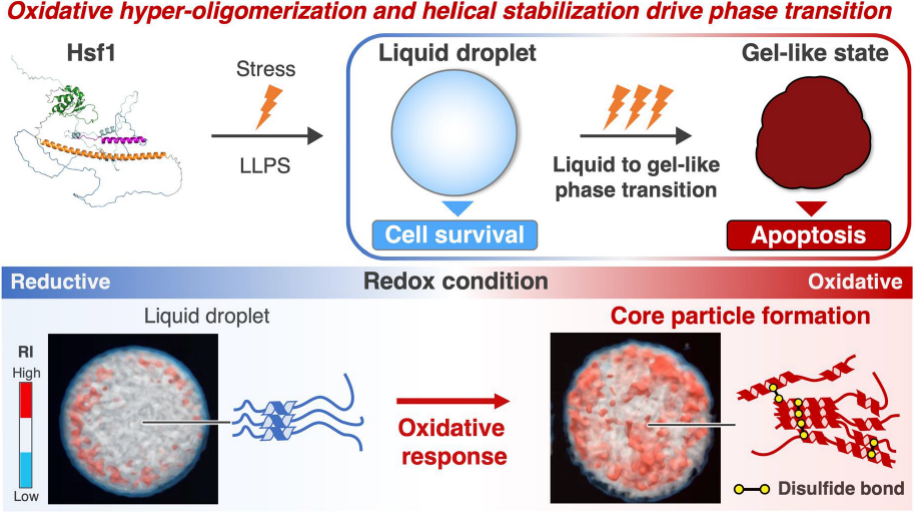

Heat shock factor 1 (Hsf1), a hub protein in the stress response and cell fate decisions, senses the strength, type, and duration of stress to balance cell survival and death through an unknown mechanism. Recently, changes in the physical property of Hsf1 condensates due to persistent stress have been suggested to trigger apoptosis, highlighting the importance of biological phase separation and transition in cell fate decisions. In this study, the mechanism underlying Hsf1 droplet formation and oxidative response was investigated through 3D refractive index imaging of the internal architecture, corroborated by molecular dynamics simulations and biophysical/biochemical experiments. We found that, in response to oxidative conditions, Hsf1 formed liquid condensates that suppressed its internal mobility. Furthermore, these conditions triggered the hyper-oligomerization of Hsf1, mediated by disulfide bonds and secondary structure stabilization, leading to the formation of dense core particles in the Hsf1 droplet. Collectively, these data demonstrate how the physical property of Hsf1 condensates undergoes an oxidative transition by sensing redox conditions to potentially drive cell fate decisions.

## Introduction

Cell-fate decisions are strategies for maintaining homeostasis in higher organisms. Under stress conditions, including heat, pH changes, and oxidation, cells either survive via increased expression of stress response proteins or activate cell death signaling.^1^ One key pathway in cell fate decisions is mediated by heat shock factor 1 (Hsf1), a transcriptional factor and stress sensor in the nucleus and cytoplasm regarded as a typical cytoprotective protein.^2^ Under proteotoxic stress conditions, Hsf1 forms membrane-less organelles, including small nuclear condensates at the heat-shock protein (HSP) gene loci and nuclear stress bodies (nSBs) at satellite III DNA repetitive sequences.^3−6^ Small nuclear condensates of Hsf1 act to upregulate molecular chaperones, including HSP70,^7−12^ and nSBs accumulate several splicing factors to promote the expression of proteins required in the recovery stage.^13,14^ Additionally, nSBs may also play a role in suppressing the production of non-HSPs to preferentially synthesize HSPs and effectively cope with stress.^15^ They resolve once the cells recover from the proteotoxic stress. Conversely, under prolonged stress, these Hsf1 condensates become a gel-like state, which may downregulate the expression of HSPs and mediate apoptosis.^16^ Thus, the physical properties of these condensates regulate Hsf1 function in stress response and can affect cell fate decisions. However, the mechanisms underlying the regulation of condensation in cells remain unknown.

The formation of membrane-less organelles in cells is often driven by biological liquid–liquid phase separation (LLPS),^17^ in which selected biomolecules accumulate and are isolated from their surroundings while maintaining internal mobility through weak interactions. Hsf1 has been suggested to undergo LLPS, with LLPS droplets transitioning into a gel-like state, both in cells and in vitro.^3,16,18^ Although the LLPS and phase transition of Hsf1 are key to understanding the stress response system, the mechanism behind LLPS regulation, more specifically the factors that govern the physical properties of Hsf1 condensate in response to the cellular environment, remain to be clarified.

In this study, we aimed to elucidate the molecular mechanisms underlying the biological phase separation and transition of Hsf1. Here, we show that oxidative conditions induce a reduction in the internal mobility of Hsf1 condensates. To investigate the redox-dependent property changes, three-dimensional (3D) refractive index (RI) imaging was used. RI imaging visualizes the internal structure of the condensates at a micrometer scale, resulting in the identification of “core particles” under oxidative conditions. To unveil the mechanism for the core particle formation, we conducted biochemical and biophysical experiments, corroborated by molecular dynamics (MD) simulations. Our results show that core particle formation is driven by the synergistic interplay between helical stabilization and hyper-oligomerization of Hsf1, depending on disulfide bond formation under oxidative conditions.

## Results

### Oxidative Environments Suppress the Internal Mobility of Hsf1 Condensates

**Figure 1:**
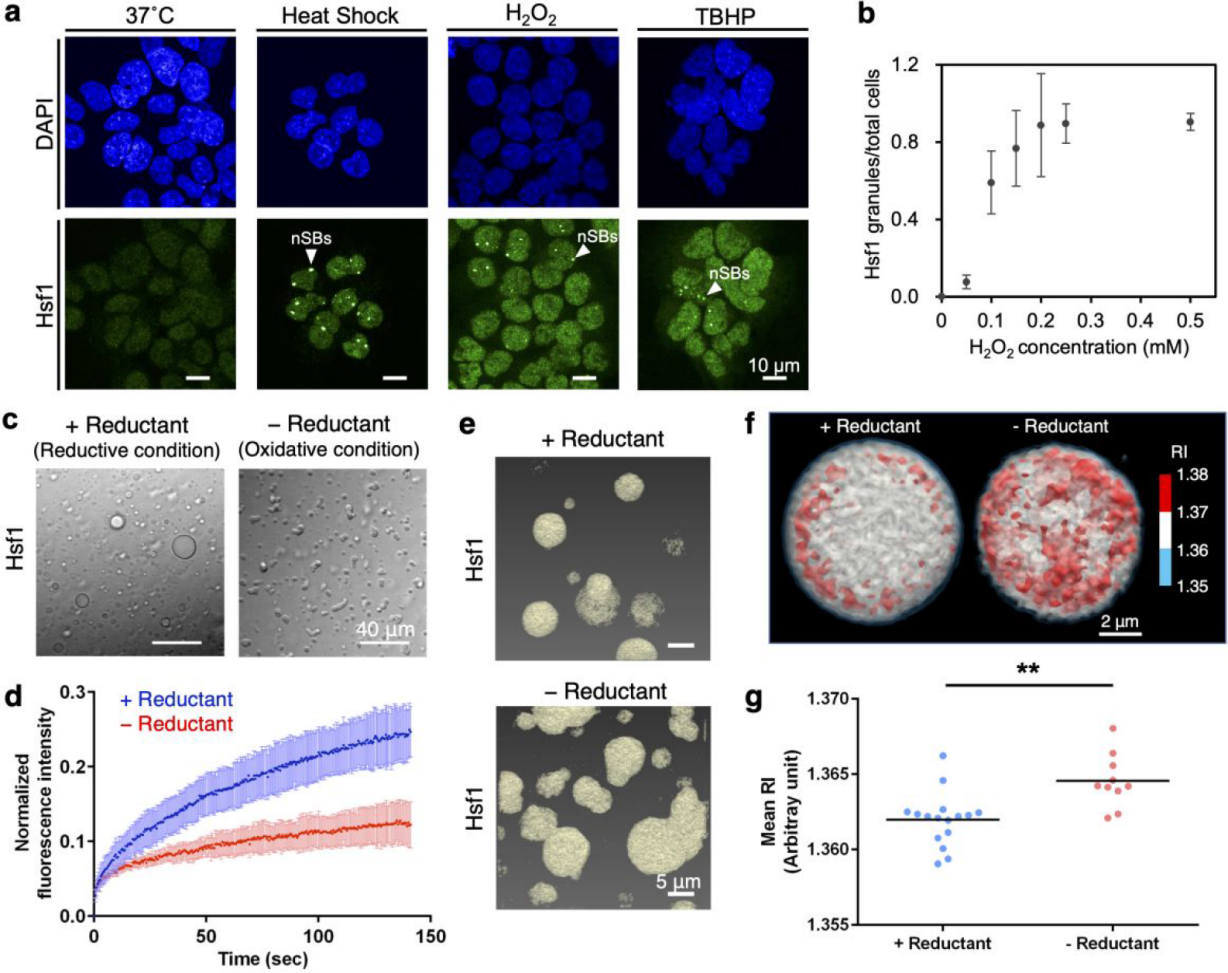
Oxidative response to lower internal-mobile state of Hsf1 droplets in vitro and in cells. (a) Confocal immunofluorescence images showing subcellular localization and foci formation of Hsf1 in HAP1 cells (scale bar, 10 μm). Cells were then costained with antibodies against HSP70 and 4′,6-diamidino-2-phenylindole (DAPI). Cells were treated with various stress conditions: 43 °C heat shock for 1 h, 1.0 mM H_2_O_2_ for 1 h, and 3.0 mM *tert*-butyl hydroperoxide (TBHP) for 1 h. (b) Number of Hsf1 foci per cell in the presence of H_2_O_2_. Cells were treated with the following H_2_O_2_ concentrations: 0, 0.05, 0.10, 0.15, 0.20, 0.25, and 0.50 mM for 2 h at 37 °C. Data are plotted as means ± s.e. of three or four independent experiments. (c) Differential interference contrast images of Hsf1 droplets in the presence of 10% (w/v) Ficoll 400. Left panels correspond to samples incubated with 10 mM DTT (+ Reductant) and the right panels correspond to samples in the absence of a reductant (− Reductant). Scale bar, 40 μm. (d) FRAP data demonstrating decreased internal-mobility of Hsf1 droplet under oxidative conditions. Bleaching event occurred at 0 s. Data are plotted as means ± s.d., with *n* = 7 independent experiments. (e) 3D RI images of Hsf1 droplets. Top and bottom panels correspond to the RI images of Hsf1 droplets in the presence or absence of 10 mM DTT (+, – Reductant), respectively. Areas with RI values greater than 1.353 are shown in yellow. (f) Quantitative analysis of RI images, measuring the mean RI of the Hsf1 droplets in the presence of 10 mM DTT (+ Reductant) and in the absence of reductant (− Reductant). (g) Scatter plot of the mean RIs of an Hsf1 droplet in the presence or absence of 10 mM DTT (+,– Reductant) with *N* = 16 (+ Reductant) and 10 (− Reductant). Each plot represents the mean RI of the average of the individual droplets. **, *p* < 0.01, statistical significance between the mean RI values in the absence or presence of the reductant.

Formation of nSBs in cultured HAP1 cells was evaluated in the presence of various types of stress, including heat shock (HS), the oxidants H_2_O_2_ or *tert*-butyl hydroperoxide (TBHP), proteasome inhibitor MG132, and the endoplasmic reticulum stress inducer tunicamycin (Figure 1a, Supplementary Figure S1a). Microscopic observation showed foci containing Hsf1 in the nucleus after HS (43 °C, 1 h) treatment, indicating that HS induced nSB formation (Figure 1a), as seen in previous studies.^4,7,19−21^ In addition to HS, the oxidants, H_2_O_2_ and TBHP, or MG132, induced nSB formation in a subset of cells (Figures 1a and S1a). As expected, tunicamycin did not induce nSB formation (Figure S1a). Notably, a dose-dependent trend was observed in nSB formation in cultured HAP1 and HeLa cells after H_2_O_2_ or TBHP treatment (Figures 1b and S1b–e), confirming the formation of nSBs under oxidative stress. Although HS and MG132 treatments do not cause direct oxidative stress, they are known to induce the generation of reactive oxygen species (ROS) in cells and make the intracellular environment more oxidative.^22^ Thus, our results highlight the relationship between Hsf1 assembly and oxidative stress.

Next, we reconstructed Hsf1 condensates in vitro in the presence of molecular crowding agents and evaluated the effects of oxidative condition on the condensates. The Hsf1 droplets under reductive conditions exhibited a round shape (Figures 1c and S2a–c). Incorporation of green fluorescent protein (GFP)-tagged Hsf1 indicated that the droplets were formed by Hsf1 molecules (Figure S2a–c). A higher concentration of Hsf1 decreased the critical concentration of the crowder during droplet formation (Figure S2d,e). Time-lapse imaging of the droplets using confocal microscopy revealed that the droplets fused within 1–2 min (Figure S2f). These observations indicate that Hsf1 forms LLPS droplets with internal mobility under reductive conditions. In contrast, the Hsf1 droplets had a distorted shape in the absence of the reducing agent, or in the presence of H_2_O_2_ corresponding to oxidative conditions (Figures 1c and S3a–c), which is characteristic of reduced internal mobility.^23^ Analysis of Hsf1 droplets through fluorescence recovery after photobleaching (FRAP) revealed suppressed fluorescence recovery under oxidative conditions compared to reduced conditions, demonstrating the decreased mobility of Hsf1 molecules in the droplets under oxidative conditions (Figure 1d). Notably, the intensity recovered to only ∼0.3 and ∼0.15 within ∼140 s under reduced and oxidized conditions, respectively. The incomplete recovery indicates the existence of immobile fractions, presumably formed through tight interactions between Hsf1 molecules. Furthermore, the reduced recovery rate under oxidized conditions suggests that immobile fractions are more abundant under oxidized conditions. Based on the findings regarding the shape and internal mobility of the droplets, we concluded that the internal mobility of Hsf1 droplets decreases under oxidative conditions.

To investigate the mechanism underlying the oxidative property change in Hsf1 droplets, RI imaging was used to investigate the internal structure of the droplets (Figure 1e–g).^24^ RI mapping revealed regions with higher RI values sporadically scattered within the droplets (Figure 1f). Given that Hsf1 is a major component (Figure S2), regions with higher RI values indicate higher concentrations of Hsf1, densely accumulating to form “core particles”. Accordingly, droplets formed under oxidative conditions exhibited a higher mean RI than those formed under reductive conditions (Figure 1g). These core particles were sparsely populated under reductive conditions and more abundant under oxidized conditions, suggesting that they contributed to the immobile fraction in the FRAP experiments (Figure 1d). Thus, the promotion of core particle formation under oxidative conditions is the key to understanding the mechanism underlying the oxidative response of Hsf1 droplets.

### Disulfide Bonds Enhance Higher-Order Oligomerization of Hsf1

**Figure 2:**
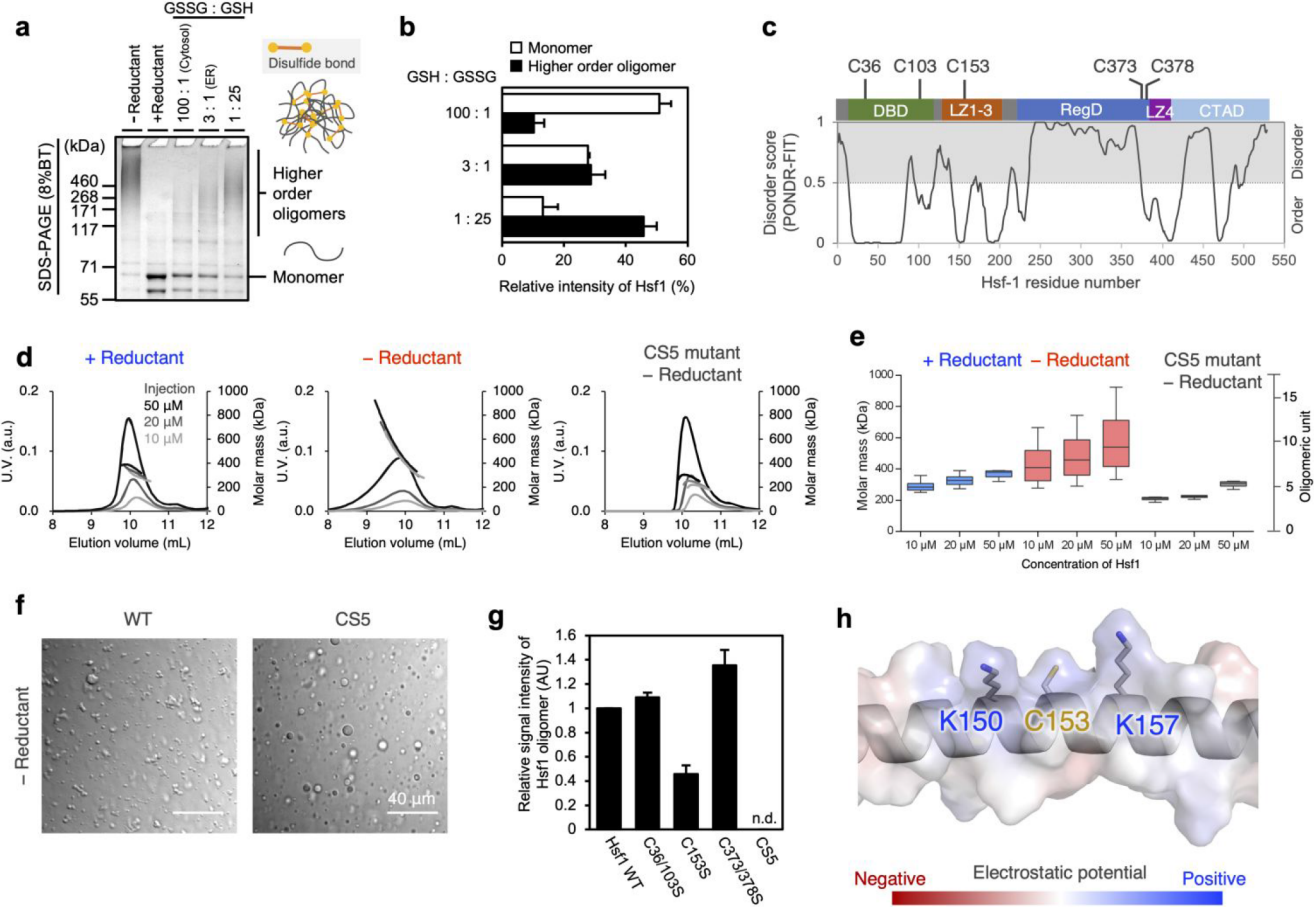
Enhanced oligomerization of Hsf1 through disulfide bonds under oxidative condition. (a) Oligomeric states of Hsf1 in redox buffers; 5 μM Hsf1 are incubated without reductant (−Reductant), with 10 mM DTT (+ Reductant), or in the presence of redox buffers (GSH: GSSG = 100:1, 3:1, 1:25) at 37 °C. Samples were quenched by addition of NEM and separated using SDS-PAGE on an 8% gel. (b) Quantification of the relative band intensities of the higher-order oligomer forms of Hsf1 compared to the band intensity of the – Reductant lane (white bar graphs), and the monomer forms of Hsf1 compared to the band intensity of the + Reductant lane (black bar graphs) in (a). Error bars correspond to the means ± s.d. of three independent experiments. (c) Domain organization, location of cysteine residues in Hsf1, and Hsf1 disorder prediction derived from the PONDR program. Following abbreviations are used: DBD, DNA-binding domain; LZ1–3, leucine-zipper domain-1-3; RegD, regulatory domain; LZ4, leucine-zipper domain-4; CTAD, C-terminal transactivation domain. (d) SEC-MALS of Hsf1 with 10 mM DTT (+ Reductant) and without reductant (− Reductant), and Hsf1 CS mutant without reductant (CS5 mutant–Reductant), injected at varying concentrations (black: 50 μM, dark gray: 20 μM, and gray: 10 μM). (e) Box and whisker plot of Hsf1 molecular mass under various conditions. (f) Differential interference contrast images of Hsf1 CS5 mutant droplets in the presence of 10% (w/v) Ficoll 400 without a reductant (− Reductant). Left panels correspond to Hsf1 WT, and the right panels correspond to Hsf1 CS5. Scale bar, 40 μm. (g) Quantification of the higher-order oligomer formation of various purified Hsf1 mutants. SDS-PAGE data (shown in Figure S7b) were used to compare the relative signal intensities of the higher-order oligomer forms in the Hsf1 mutant lanes with the signal intensity in the WT lane. Error bars correspond to the means ± s.d. of three independent experiments. n.d.: not detectable. (h) Close-up view of the structure around Cys153 with an electrostatic potential map of Hsf1, generated using the PyMol plugin (vacuum electrostatics).

The increase in core particles in the Hsf1 droplet under oxidative conditions suggests a tighter molecular assembly of oxidized Hsf1. Nonreducing sodium dodecyl sulfate-polyacrylamide gel electrophoresis (SDS-PAGE) showed that Hsf1 forms disulfide-linked oligomers in the absence of a reductant (Figure 2a). Furthermore, the population of disulfide-linked oligomers was evaluated under varying redox conditions, including those corresponding to the endoplasmic reticulum environment with a ratio of reduced glutathione (GSH) to oxidized glutathione (GSSG) of 3:1 and the cytosolic environment with a GSH:GSSG ratio of 100:1 (Figure 2a, b).^25^ The data showed that the fraction of higher-order oligomers increased and that of monomers decreased as the redox conditions became more oxidized (Figure 2a, b). Since the redox conditions in the cytosol vary depending on the external or local conditions,^26−28^ these data imply that Hsf1 can sense the redox environment in the cell by modulating the abundance of disulfide-linked oligomers. Formation of these oligomers by Hsf1 was also observed in HAP1 cells after treatment with H_2_O_2_ or TBHP, in a dose-dependent manner (Figure S4).

Hsf1 contains five conserved cysteine residues (Figures 2c and S5) that are thought to participate in intermolecular disulfide bonding. We designed an Hsf1 CS5 mutant in which all five cysteine residues were replaced with Ser residues. The oligomeric state of the mutants in dilute solution was evaluated using size-exclusion chromatography with multiangle light scattering (SEC-MALS) under reductive and oxidative conditions (Figure 2d,e). Under reductive conditions, the SEC elution profile of Hsf1 wild-type (WT) was highly symmetric (Figure 2d), with a median molar mass of 379 kDa at an injection concentration of 50 μM, corresponding to approximately a 7-mer (Figure 2d,e). In contrast, under oxidative conditions, the elution peak became asymmetric and the median molar mass increased to 540 kDa, corresponding approximately to a 9-mer (Figure 2d,e). Furthermore, a significant population of Hsf1 was found to be made up of larger oligomers, up to approximately 16-mer, under oxidative conditions (Figure 2d,e). Hsf1 CS5 mutants under oxidative conditions did not form higher-order oligomers, and their median molar mass was 308 kDa, corresponding to an approximately 5-mer (Figure 2d,e). The addition of DTT to Hsf1 CS5 mutants did not affect the molar mass, suggesting that DTT is involved only in the deformation of disulfide bonds and does not affect the noncovalent interaction between Hsf1 protomers (Figure S6a,b). These SEC-MALS data suggest that Hsf1 oligomerization is mediated by both noncovalent interactions and disulfide bonds. To evaluate the contribution of intermolecular disulfide bonds to oligomerization, SDS-PAGE was performed on Hsf1 samples incubated in the presence or absence of a reductant and quenched with N-ethylmaleimide (NEM). NEM-quenched SDS-PAGE showed that disulfide-linked higher-order oligomers (>460 kDa) were detected only in Hsf1 WT prepared under oxidative conditions (Figure S7a), indicating the formation of disulfide bonds in the oligomer. Microscopic observation of Hsf1 CS5 droplets revealed that the droplets of the CS5 mutant remained spherical even under oxidative conditions (Figures 2f and S3a–c), corroborating the idea that disulfide-linked higher-order oligomers in an oxidative environment lead to the formation of core particles.

Critical cysteine residues involved in the formation of disulfide-linked oligomers were identified by point mutations in the cysteine residues. Nonreducing SDS-PAGE analysis showed that the C153S mutation lacking cysteine residues in the leucine-zipper domain (LZ1–3) induced the most significant reduction in oligomeric components among the cysteine mutants tested (Figures 2g and S7b), suggesting that Cys153 plays a key role in intermolecular disulfide bond formation. Cys153 was accompanied by the basic amino acids Lys150 and Lys157 (Figure 2h), whose positive charges can enhance thiol reactivity.^29,30^ The impact of disulfide bonds on noncovalent interactions was evaluated through analytical SEC at varying concentrations. The CS5 mutant showed the largest change in elution volume with decreasing protein concentration (Figure S8a,b), suggesting that the formation of disulfide bonds strengthened the noncovalent interactions. Notably, the C153S mutant showed the largest concentration-dependent change in elution volume among the selected mutants tested, suggesting that the disulfide bond via Cys153 makes the most significant contribution to noncovalent interactions.

### Secondary Structure Stabilization Promotes the Assembly of Hsf1

**Figure 3:**
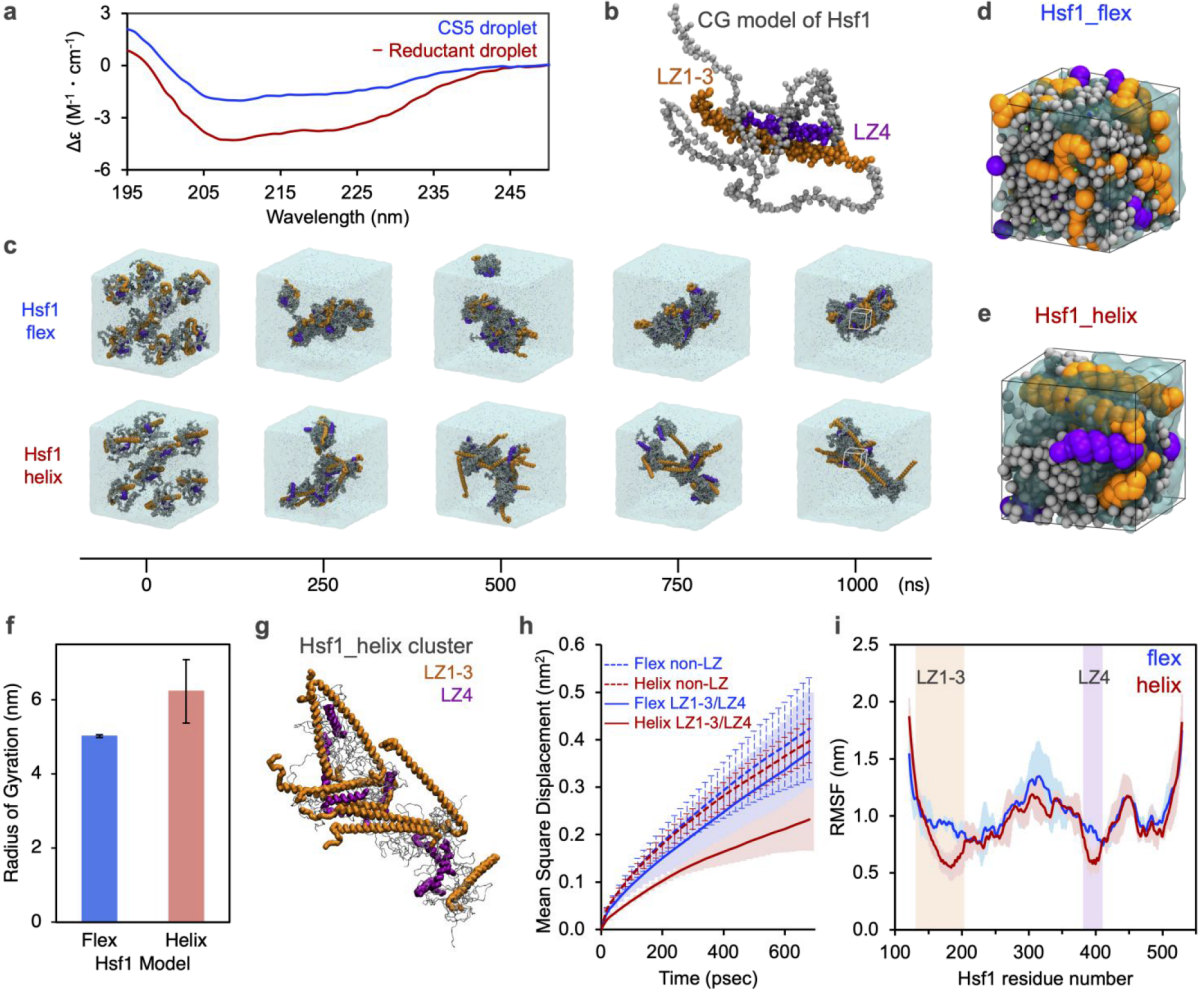
Oxidative α-helix stabilization promotes the assembly of Hsf1. (a) SRCD spectra of 40 μM each of Hsf1 WT and CS5 in the presence of 18.75% (w/v) dextran 200. (b) Coarse-grained (CG) configurations of the Hsf1 structure derived from AlphaFold2, where the DBD region (residues 1–121) is excluded. MARTINI CG model relies mainly on a four-to-one mapping scheme; that is, on average, four heavy atoms and the associated hydrogen atoms are mapped to one bead. LZ1–3 (residue numbers: 121–207) of Hsf1 is colored orange, LZ4 (residue numbers: 380–411) is colored purple, and the rest is colored gray. (c) Time evolution of Hsf1_helix and Hsf1_flex in 150-mM KCl aqueous solution. K^+^ and Cl^–^ charged beads are shown as small dots in blue and green, respectively, and water beads represent the surface. (d,e) Close-up view of the internal structure of the Hsf1 flex (d) and helix (e) clusters, with water depicted by the surface model in cyan, showing that the Hsf1 cluster remained well hydrated. (f) Gyration radius of the Hsf1 cluster. Higher value for Hsf1_helix indicates that the cluster is elongated and nonspherical owing to the steric hindrance of the helices. Error bars correspond to the means ± s.d. of three independent simulations. (g) Snapshot of the Hsf1 cluster in the Hsf1_helix model at 1000 ns. The LZ1–3 (orange) and LZ4 (purple) helices are shown in a thickened licorice representation, whereas the others are shown in a line representation. (h) Analysis of Hsf1 diffusion in complex clusters. Short-term mean square displacements of the main-chain beads in the LZ and non-LZ domains were compared between the Hsf1_flex and Hsf1_helix models. Smaller slope of the LZ domain in the Hsf1 helix model indicates a lower diffusivity of the LZ helices compared to that of the coiled LZ domain in the Hsf1_flex and non-LZ domains. Error bars correspond to the means ± s.d. of three independent simulations. (i) Root-mean-square fluctuation for each amino acid residue of the Hsf1_flex (blue) and Hsf1_helix (red) models. Helical regions in LZ1–3 and LZ4 are highlighted in orange and purple, respectively. Error bars correspond to the means ± s.d. of three independent simulations.

Disulfide bonds often stabilize protein structures. We hypothesized that the structural effects of Hsf1 disulfide bonds alter the intermolecular interactions. To evaluate the secondary structure of Hsf1 in the droplets, we used synchrotron-radiation circular dichroism (SRCD), which combines a 50-μm ultrathin cell and a detector right behind the cell. This eliminates the impact of the light scattering from turbid sample solutions including the droplets.^31^ The SRCD spectra of the Hsf1 WT droplet formed in the presence of dextran without the reductant showed minimum ellipticity at 207 nm, characteristic of high α-helical content (Figures 3a and S9a). In contrast, the negative ellipticity in the 205–230 nm region decreased in the Hsf1 CS5 droplet (Figures 3a and S9b). The α-helical content calculated from these SRCD spectra using the BeStSel program^32^ was 24.4% in WT and 13.2% in CS5, indicating that disulfide bonds stabilized the helical structure of Hsf1 in the droplet. The α-helical content in the Hsf1 WT droplet showed good agreement with the calculated α-helical content of 28.4%, as estimated from the Alphafold2 model including α-helices in the DBD, LZ1–3, and LZ4 (Figure S9c,d). Although the monomeric structure of Hsf1 other than DBD has not been solved, helix formation of LZ1–3 and LZ4 is supported by reasonably high predicted local distance difference test (pLDDT) score and is consistent with disorder prediction (Figures 2c and S9d).

To assess the effect of helical structure stabilization on the internal mobility of Hsf1 droplets at the molecular level, we performed coarse-grained (CG) MD simulations. A CG model of the Hsf1 structure without DBD (a.a. 121–529) derived from Alphafold2 was constructed (Figures 3b and S9d), and MD simulations were performed using two different Hsf1 models: “Hsf1_helix” and “Hsf1_flex”. In the Hsf1_helix model, the secondary structures of the LZ1–3 (a.a. 121–209) and LZ4 (a.a. 379–411) were preserved during the calculation, whereas in the Hsf1_flex model, no such restrictions were placed on the secondary structure (Figures 3c and S10). Both calculations showed that the Hsf1 molecules assembled and formed clusters within ∼900 ns (Figures 3c and S11c,d). Notably, water molecules were contained in the Hsf1 clusters (Figure 3d,e), suggesting a loose interaction between the Hsf1 molecules in the cluster, as often seen in the LLPS condensates of intrinsically disordered proteins.^33^ A reference simulation of the globular folded-protein bovine serum albumin (BSA) revealed that the surface area of BSA underwent minimal changes throughout the simulation, indicating that the BSA molecules remained dispersed (Figure S11a–c). The cluster formed by Hsf1_helix had an elongated shape, whereas that formed by Hsf1_flex had a more compact globular shape. The difference in the shapes of the clusters was also highlighted by the gyration radius (*R*_g_), which was higher for Hsf1_helix than for Hsf1_flex (Figure 3f). Next, we evaluated the mobility of the helical structure regions, LZ1–3 and LZ4, that are responsible for the regulation of oligomerization.^34^ The mean square displacement (MSD) and the per-residue root-mean-square fluctuation (RMSF) for Hsf1 after 900 ns of simulation showed that the LZ1–3/LZ4 regions in the Hsf1_helix cluster had reduced mobility compared with those in the Hsf1_flex cluster (Figure 3g–i). Focusing on the region where the helix structure was clustered in Hsf1_helix, the reduced mobility of the LZ1–3/LZ4 regions in this cluster was also highlighted by the analysis of the distance between Leu residues located on the helix bundle (Figure S12). The distance trajectory between Leu395 on LZ4 and the three closest Leu residues in the final structure revealed that these distances were closer and more stable in the Hsf1_helix cluster than in the Hsf1_flex cluster (Figure S12), indicating a more stable bundle structure formed by helix–helix interactions in the Hsf1_helix cluster.

To further link the helix-to-helix interactions with Hsf1 cluster fluidity, we constructed a two-dimensional free energy surface (2D FES) using two collective variables, specifically, the distance between Leu residues and the gyration radius (Figure S13). From the energy basins identified in the 2D FES, k-means clustering was applied to extract representative snapshots of each metastable state. We integrated principal component analysis (PCA) to further validate the clustering results obtained using k-means. PCA was performed on the trajectory data from the energy basins, and the first two principal components were used to visualize the main structural variations in the system. The clustering results were then projected onto this reduced space, showing a consistent grouping of similar conformations in both the PCA and k-means results (Figure S13c). This confirms the robustness of the identified metastable states and structural stability across the simulated systems. In conclusion, the SRCD and MD data demonstrate that helical stabilization via disulfide bonds under oxidative conditions drives a tighter assembly between Hsf1 molecules in droplets.

## Discussion

Despite the importance of the Hsf1 condensate and its physical properties in stress response and cell fate decisions, the scarcity of biochemical and biophysical information has impeded the understanding of its detailed mechanism. In the present study, we found that nSBs containing Hsf1 were formed in cultured cells in response to oxidative stress (Figure 1a,b). Microscopic observation of the droplets formed by purified Hsf1 demonstrated that oxidative conditions reduced the internal mobility of the Hsf1 droplet (Figure 1c,d). This observation suggests that oxidation contributes to the phase transition in nSBs to a gel-like state under continuous stress,^16^ where the cytosol becomes oxidized due to excessive ROS generation.^35−37^ Since this phase transition leads to apoptosis,^16^ our data suggest that the oxidative response of Hsf1 is a key factor in the continuous stress response and in cell fate decisions. Furthermore, given that ROS are often used in interorganelle communication during stress response,^38,39^ the oxidative response of Hsf1 is essential for understanding the cellular stress response network.

**Figure 4:**
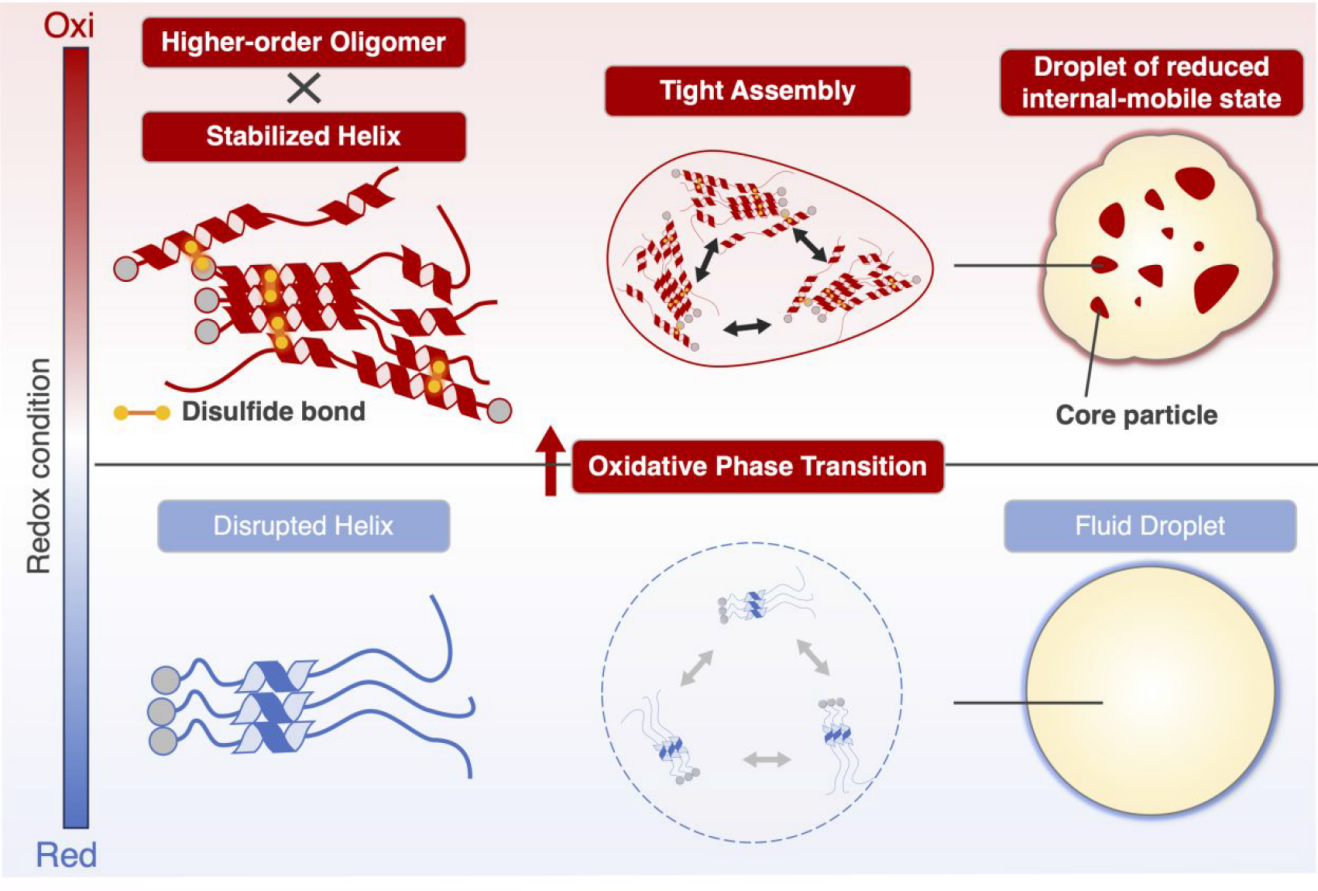
Proposed transition mechanism of Hsf1 droplets. Hsf1 condensate undergoes a transition to lower internal-mobile state in a redox-dependent manner. Disulfide bond formation promotes higher-order oligomerization and stabilization of helix. These events enhance the assembly between oligomers, resulting in core particle formation.

RI imaging demonstrated that the Hsf1 molecules were heterogeneously distributed in the droplets, especially under oxidative conditions, forming core particles with a higher density (Figure 1f,g). Larger disulfide-linked oligomers of Hsf1 formed under oxidative conditions, as revealed by SEC-MALS and SDS-PAGE (Figure 2), were expected to have an increased number of interaction points with other oligomeric units, resulting in a higher Hsf1 density, forming core particles, and consequently, reduced mobility of Hsf1 molecules in the gel-like droplets (Figure 4). SDS-PAGE data showed that the population of disulfide-linked oligomers of Hsf1 was modulated in response to redox potentials (Figure 2a,b,g), indicating that Hsf1 responds to cellular redox changes by modulating the oligomers. Among the five cysteine residues in Hsf1, C153 was found to have the most significant contribution to oxidative oligomerization (Figures S7 and S8). Cys153 is particularly well-conserved in monkeys and mice, which are taxonomically close to humans (Figure S5a,b), implying that Cys153 of Hsf1 was acquired during evolution to reinforce the stress response under oxidative conditions. Disulfide bonds stabilized the secondary structure of Hsf1 in the droplet, as revealed by SRCD, leading to a tighter assembly of Hsf1 oligomers (Figure 3). A reduction in mobility inside the droplet by stabilizing the helical structure was also demonstrated for a transactive response DNA-binding protein of 43 kDa (TDP-43).^40^ Given the frequent appearance of transient helical strictures in intrinsically disordered proteins and those involved in LLPS,^41,42^ secondary structure formation in the disordered region can be a strategy to regulate droplet properties in response to environmental change.

Taken together, the proposed mechanism of the transition of Hsf1 droplets in response to oxidative conditions is as follows: under oxidative conditions, Hsf1 forms intramolecular and intermolecular disulfide bonds. These disulfide bonds simultaneously promote the formation of higher-order oligomers and stabilization of the helical structure, resulting in increased interaction points and tighter interactions between oligomers, and consequently, in the formation of core particles in the droplets (Figure 4). The increased core particle formation results in higher density and lower mobility inside the droplet. This mechanism of the redox-dependent transition of Hsf1 condensates can explain the phase transition of nSBs to a gel-like state and small Hsf1 condensates under continuous stress, potentially mediating cell fate decisions.

Furthermore, the data obtained in this study revealed that the Hsf1 oligomers were larger than those described in previous schemes.^2,12,43^ Conventional models describe that Hsf1 exists as a monomer in the cytoplasm under normal conditions, whereas stress conditions induce trimerization, leading to its translocation to the nucleus.^11,12,43,44^ Alternatively, several previous studies using electrophoresis and gel filtration column chromatography have indicated that Hsf1 forms larger oligomers than trimers.^8,34,45,46^ Our quantitative analysis of oligomers in solution using SEC-MALS demonstrated that Hsf1 forms oligomers with a median mass of a 7-mer under reductive conditions and 9-mer under oxidative conditions, with fractions reaching up to approximately a 16-mer under oxidative conditions (Figure 2d,e). Oxidative conditions such as “hyper-oligomerization” can be particularly important in driving the state transition of Hsf1 droplets.

Because nSBs comprise RNA polymerase II and bromodomain-containing protein (BRD4),^4^ the redox-dependent phase transition of Hsf1 is expected to perturb the activity of these components. Both RNA polymerase II and BRD4 are involved in the transcription of satellite III RNA^47,48^ and the assembly of other nSBs components, as well as in the associated DNA/RNA metabolism, biosynthesis, stress response, and cell cycle.^13,14^ Hsf1 may affect the regulation of these cellular events through redox-dependent phase transitions, modulating the intracellular stress response system and influencing cell fate decisions.

## Materials and Methods

### Expression and Purification of Protein Samples

The human Hsf1 and Hsf1-GFP expression constructs were cloned into a pET21b vector (Cat. No. 69741-3CN; Novagen, Madison, Wisconsin, USA) and fused to GB1-His6 tags at the HRV3C N-terminus of the protease cleavage site. Hsf1 C36S/C103S, C153S, C373S/C378S, and CS5 mutants (C36S/C103S/C153S/C373S/C378S) were constructed by site-directed mutagenesis using the PrimeSTAR Mutagenesis Basal Kit (Cat. No. R046A; Takara Bio, Shiga, Japan). All expression constructs were transfected into BL21(DE3) cells. With respect to the samples, cells were grown in Luria–Bertani medium at 37 °C in the presence of ampicillin (50 μg mL^–1^). Subsequently, protein expression was induced by adding 0.5 mM isopropyl-β-D-1-thiogalactopyranoside at OD_600_ ∼ 0.6, followed by 12–16 h of incubation at 18 °C. The cells were harvested at OD_600_ ∼ 3.0, resuspended in lysis buffer containing 50 mM Tris-HCl (pH 8.0) and 500 mM NaCl, disrupted in a sonicator, and centrifuged at 18,000 rpm for 30 min. The supernatant fraction containing Hsf1 was also purified using a Ni-NTA Sepharose column (Cat. No. 30210; QIAGEN, Hilden, Germany). Additionally, the GB1-His6 tag was removed using HRV3C protease at 4 °C (incubation for 16 h), after which the cleaved Hsf1 was applied to a HiTrap Q HP anion exchange column (Cat. No. 17115401; Cytiva, Tokyo, Japan), pre-equilibrated with 25 mM HEPES/NaOH (pH 7.5), 5 mM MgCl_2_, 10% glycerol, and 20 mM NaCl, and eluted with a linear gradient of 20–500 mM NaCl. Hsf1 oligomers and monomers were eluted separately through anion-exchange purification. Hsf1 oligomers were further purified by gel filtration using a Superdex 200 pg 16/600 column (Cat. No. 28989335; Cytiva), and equilibrated with a solution containing 25 mM HEPES/KOH (pH 7.2) and 150 mM KCl. Finally, protein concentrations were determined spectrophotometrically at 280 nm using the corresponding extinction coefficients.

### Confocal Microscopy

To prepare Hsf1 droplets, 45 μM Hsf1 and 5 μM Hsf1-GFP were incubated in the presence/absence of 10 mM DTT, 10% (w/v) Ficoll 400 (Cat. No. 16006-92; Nacalai Tesque, Kyoto, Japan), and dextran 200 (Cat. No. 10927-12; Nacalai Tesque) or PEG 8000 (Cat. No. HR2-515; Hampton Research, Journey Aliso Viejo, CA, USA). Fluorescence images of Hsf1 droplets were obtained using a confocal microscope (FV1200, Olympus, Tokyo, Japan) equipped with a UPLSAPO 40 × 2 objective lens (NA 0.95).

### Fluorescence Recovery after Photobleaching

FRAP experiments were performed on in vitro droplets formed by Hsf1 that were mixed with Hsf1-GFP using the 473 nm laser line of a confocal microscope (FV1200, Olympus), equipped with a UPLSAPO 40 × 2 objective lens (NA 0.95). For each droplet, either the whole droplet or a specific spot (diameter of 5 μm) was bleached at 80% transmission (50 mW laser power) at 20th and 21st frames, after which postbleach time-lapse images were collected (0.5 s frame rate, 180 frames). The resulting images were analyzed as follows: a 5 μm diameter region of interest (ROI) was placed on the bleached whole droplet or spot. The fluorescence intensity of the ROI was calculated using FV10-ASW (Olympus). The postirradiation fluorescence intensity was normalized using the difference between the intensity before irradiation and that of the first frame immediately after irradiation. Finally, the recovery of the postirradiation fluorescence intensity was analyzed using Prism 5 (GraphPad Software, San Diego, CA, USA).

### SEC-MALS Experiments

SEC-MALS was measured using DAWN HELEOS8+ (Wyatt Technology Corporation, Santa Barbara, CA, USA), a high-performance liquid chromatography pump LC-20AD (Shimadzu, Kyoto, Japan), refractive index detector RID-20A (Shimadzu), and UV–vis detector SPD-20A (Shimadzu), located downstream of the Shimadzu liquid chromatography system and connected to a Bio SEC-5, 1000 Å gel filtration column (Cat. No. AG5190-2536; Agilent Technologies, Santa Clara, CA, USA). Differential RI (Shimadzu) downstream of MALS was used to determine protein concentrations. The running buffer comprised 25 mM HEPES/KOH (pH 7.2) and 150 mM KCl. A total of 100 μL of the sample was injected at a flow rate of 1.0 mL min^–1^. The data were analyzed using ASTRA version 7.0.1 (Wyatt Technology Corporation). Molar mass analysis was performed over half the width of the top height of the UV peak, after which box and whisker plots were created using Prism 5 (GraphPad Software).

### RI Imaging

3D quantitative phase images of Hsf1 droplets were obtained using a commercial holotomography instrument (HT-2H, Tomocube Inc., Daejeon, Korea), based on Mach–Zehnder interferometry and equipped with a digital micromirror device. The coherent monochromatic laser (λ = 532 nm) was divided into two paths, a reference and a sample beam, using a 2 × 2 single-mode fiber coupler. The 3D RI maps were then visualized using commercial software (TomoStudio, Tomocube Inc.). The RI reference buffer consisted of 25 mM HEPES/KOH (pH 7.2), 150 mM KCl, and 5% (w/v) Ficoll 400 (200 μL). Moreover, 75 μM Hsf1 that was incubated in the presence/absence of 10 mM DTT and 5% (w/v) Ficoll 400 was added to 50 μL of the RI reference buffer, after which RI images of the Hsf1 droplets were measured. To determine the mean RI of the Hsf1 droplets, images of the xy-plane slices at the center of the droplet were initially exported, with RI values ranging 1.349–1.402, displayed as a gray-gradient color image. To quantify the RI inside the Hsf1 droplets, 3–8-μm droplets were selected and the mean RI of each droplet was then determined to generate a scatter plot. Finally, the exported images were analyzed using the ImageJ software (National Institutes of Health, Bethesda, MD, USA), and the gradients were numerically converted into 256 steps. The mean RI of each droplet was determined using Prism 5 (GraphPad Software). Data were analyzed using Welch’s *t* test.

### Gel-Based Analysis of Hsf1 Redox States under Oxidative or Reductive Conditions

Hsf1 (50 μM) was incubated with a reaction buffer containing 25 mM HEPES-KOH (pH 7.2) and 150 mM KCl in the absence or presence of 10 mM DTT at 20 °C for 1 h. To prepare NEM-quenched samples, a 12-μL aliquot was collected from the reaction mix, after which 48 μL of 90 mM NEM (Cat. No. 15512-24; Nacalai Tesque) was added to quench the reaction. Additionally, 60 μL of Laemmli 4 × sodium dodecyl sulfate (SDS)-sample buffer^49^ were added to these samples (total volume: 120 μL). Samples were then separated by SDS-PAGE on 8% bis-tris gels (Cat. No. NW00080BOX; Thermo Fisher Scientific, Waltham, MA, USA) for comparison of WT and CS mutants or on 4–12% bis-tris gels (Cat. No. NW04120BOX; Thermo Fisher Scientific) to compare the various cysteine mutants. To prepare reducing samples, a 12-μL sample aliquot, 48 μL distilled water, and 60 μL SDS-sample buffer containing 5% (v/v) β-mercaptoethanol (Cat. No. 15512-24; Nacalai Tesque) was added to reduce and denature the samples.

### Gel-Based Analysis of Hsf1 Oligomeric States in Redox Buffers

The redox buffers were prepared by mixing reduced glutathione (Cat. No. 17050-14; Nacalai Tesque) and oxidized glutathione (GSSG; Cat. No. 06440-31; Nacalai Tesque) in the following final concentration ratios: 9.8 mM GSH and 0.1 mM GSSG (100:1), 6.0 mM GSH and 2.0 mM GSSG (3:1), or 0.2 mM GSH and 4.9 mM GSSG (1:25). Hsf1 (5 μM) was incubated at 37 °C for 1 h with a reaction buffer containing 50 mM Tris-HCl (pH 8.0) and 150 mM KCl in the absence/presence of 10 mM DTT, or in the presence of a redox buffer. A 40-μL aliquot was taken from the reaction mix and 10 μL of 40 mM NEM (Nacalai Tesque) was added to quench the reaction. In addition, 50 μL of Laemmli 4 × SDS-sample buffer^49^ was added to the samples (total volume: 100 μL), which were separated by SDS-PAGE using 8% bis-tris gels (Thermo Fisher Scientific).

### Cell Culture

HAP1 cells were obtained from Horizon Discovery (Cambridge, UK), cultured, and maintained at 37 °C and 5% CO_2_ in Iscove’s modified Dulbecco’s medium containing l-Gln and HEPES (Cat. No. 11506-05; Nacalai Tesque) and 10% fetal bovine serum (FBS; Cat. No. S-FBS-NL-015; Serana, Brandenburg, Germany). HeLa cells were obtained from RIKEN BRC, which is participating in the National Bio-Resource Project of MEXT, Japan. The HeLa cells were cultured and maintained at 37 °C and 5% CO_2_ in RPMI1640 medium (Cat. No. 30264-56; Nacalai Tesque) with 10% FBS (Cat. No. F7254; Sigma-Aldrich, Tokyo, Japan).

### Immunofluorescence under Various Stress Conditions

To analyze the formation of Hsf1 foci in HAP1 cells, the cells were seeded on coverslips (Cat. No. C018001; Matsunami Glass Industry, Osaka, Japan) pretreated with ε-poly-l-lysine coating solution (Cat. No. SPL01, Cosmo Bio Co.). After a 1-day incubation, the cells were treated with fresh culture medium (37 °C control) or prewarmed medium at 43 °C (HS) for 1 h using a water bath; with medium in the absence/presence of 0.5 and 1.0 mM H_2_O_2_ or 1.0 and 3.0 mM TBHP for 1 h; or with medium containing 0.1% (v/v) DMSO in the absence/presence of 2 μM MG132 (Cat. No. CS-0471; ChemScene, Monmouth Junction, NJ, USA) or 2 μg/mL tunicamycin (Cat. No. 202-08241; Fujifilm, Wako Pure Chemical) for 2 h. The treated cells were washed twice with phosphate-buffered saline (PBS; 10 mM Na_2_HPO_4_, 1.76 mM KH_2_PO_4_, 137 mM NaCl, 2.7 mM KCl) at room temperature (RT) and fixed with a 4% paraformaldehyde phosphate buffer solution (Cat. No. 09154-58; Nacalai Tesque) for 15 min. The fixed cells were washed four times with PBS at RT and permeabilized with PBS containing 0.1% TritonX-100 (Cat. No. 12967-32; Nacalai Tesque) for 15 min, and then blocked with PBS containing 2% FBS for 1 h at RT. Next, the cells were incubated at 4 °C with antibodies against Hsf1 (Cat. No. 4356; Cell Signaling Technology, Danvers, MA, USA; 1:500) and HSP70 (Cat No. sc-24; Santa Cruz Biotechnology, Santa Cruz, CA, USA; 1:200), and then diluted overnight in PBS containing 2% FBS. Following incubation, the cells were washed thrice with PBS at RT, and then incubated at 4 °C for 1 h with CF488A-conjugated donkey antirabbit IgG (Cat No. 20015-1; Biotium, Fremont, CA, USA; 1:2000) and CF568-conjugated goat antimouse IgG (Cat No. 20101-1; Biotium; 1:2000) as secondary antibodies, diluted in PBS containing 2% FBS. Finally, the cell nuclei were stained with DAPI (Cat. No. 19178-91; Nacalai Tesque; 1:10000) and fluorescence images were obtained using a confocal microscope (FV1200, Olympus) equipped with a 60× silicon oil-immersion objective lens (UPLSAPO60XS2, Olympus; NA 1.30).

To analyze the Hsf1 foci in HeLa cells under oxidative stress, the cells were seeded on coverslips (Matsunami Glass Industry). After a 1-day incubation period, the cells were treated with fresh medium in the absence/presence of 0.05, 0.1, 0.15, 0.20, 0.25, and 0.50 mM H_2_O_2_ (Cat. No. 084-07411; Fujifilm Wako Pure Chemical) for 2 h at 37 °C and 5% CO_2_. The treated cells were fixed with a 4% paraformaldehyde phosphate buffer solution (Cat. No. 163-20145; Fujifilm Wako Pure Chemical) for 15 min at RT. The cells were then washed twice with PBS (Cat. No. 05913; Nissui Pharmaceutical, Tokyo, Japan) and permeabilized in PBS containing 0.5% TritonX-100 (Cat. No. T9284; Sigma-Aldrich) for 10 min at 4 °C. Subsequently, they were washed twice with PBS again. Next, the cells were blocked with blocking buffer (PBS containing 0.01% Tween20 [Cat. No. P1379; Sigma-Aldrich] and 10% FBS [Cat. No. S1560-500; Biowest, Nuaille Pays de la loire, France]) for 1 h at RT. Then, they were treated with a blocking buffer containing Rabbit anti-Hsf1 antibody (Cat. No. 4356S; Cell Signaling Technology, Danvers, MA, United States; dilution 1:1000) at 4 °C for 18 h. The cells were washed thrice with PBS containing 0.2% Tween20, and then treated with a blocking buffer containing Alexa Fluor 647 goat antirabbit IgG [H+L] (Cat. No. A21244; Sigma-Aldrich; dilution 1:1000) for 1 h at RT. The treated cells were again washed thrice with PBS containing 0.2% Tween20. The cells were then incubated in PBS containing DAPI solution (Cat. No. 340-07971; Dojindo, Kumamoto, Japan; dilution 1:10000) for 5 min at RT. Fluorescence images were obtained using a confocal microscope (FV1000, Olympus) equipped with a 60× oil-immersion objective lens (UPLSAPO60XO, Olympus; NA 1.35).

### Immunoblotting under Oxidative Conditions

To investigate Hsf1 oligomerization under oxidative stress, HAP1 cells (5.0 × 10^4^ cells) were plated in a 6-well plate. After a 3-day incubation, the cells were treated with cell culture medium in the absence or presence of 0.5 and 1.0 mM H_2_O_2_ or 1.0 and 3.0 mM TBHP (Cat. No. 026-13451; Fujifilm Wako Pure Chemical, Osaka, Japan). The treated cells were incubated for an additional 1 h and then washed twice with PBS at 37 °C, following which they were acid-quenched with ice-cold 10% (w/v) TCA (Cat. No. 34637-14; Nacalai Tesque).^50^ The harvested cells were centrifuged at 15,000 × *g* for 2 min at 4 °C. The precipitates were washed and sonicated twice in ice-cold acetone using a Bioruptor UCD-300 (Tosho Denki, Yokohama, Japan) before being dissolved in an SDS-sample buffer containing NEM (2% [w/v] SDS, 100 mM Tris-HCl [pH 6.8], and 50 mM NEM). The samples were separated by SDS-PAGE using 6% tris-glycine gels (Cat. No. XP00060BOX; Thermo Fisher Scientific) and transferred to PVDF membranes (Cat. No. IPVH07850; Merck Millipore, Darmstadt, Germany) using eBlot L1 (GenScript, Piscataway, NJ, USA). The membranes were blotted with an anti-Hsf1 antibody (Cat. No. 4356, Cell Signaling Technology; 1:1000), diluted in Signal Enhancer HIKARI Solution A (Cat. No. 02270-81; Nacalai Tesque), and a secondary antirabbit antibody (Cat. No. 711-035-152; Jackson ImmunoResearch Laboratories, West Grove, PA, USA; 1:10000), diluted in Signal Enhancer HIKARI Solution B. Chemiluminescent signals were visualized using Chemi-Lumi One Ultra (Cat. No. 11644-24; Nacalai Tesque) and scanned using ImageQuant LAS 4000 mini (Fujifilm, Tokyo, Japan) and Amersham ImageQuant 800 (Cytiva). The total protein concentration was measured using Coomassie brilliant blue staining (Figure S4c). The signal intensities were analyzed using ImageJ/Fiji^51^ and Microsoft Excel. Chemiluminescence signal intensities were normalized to the total protein signal from the Coomassie brilliant blue staining. The relative intensities corresponding to higher-order oligomeric Hsf1 were calculated by comparing them to the control lane on each membrane. Data were analyzed using one-way analysis of variance, followed by the Tukey–Kramer test.

### Disorder Predictions

Intrinsically disordered Hsf1 regions were predicted using the “VSL2” algorithm of the “Predictor of Natural Disordered Regions” (PONDR, http://www.pondr.com/).

### Synchrotron-Radiation Circular Dichroism

The SRCD spectra of Hsf1 and CS5 (40 μM) in the presence of dextran 200 were recorded from 263 to 175 nm using a vacuum-ultraviolet circular dichroism (VUVCD) spectrophotometer at the Hiroshima Synchrotron Radiation Center (HiSOR) and an assembled optical cell with a 50-μm path length Teflon spacer. The measurements were taken at 25 °C with 10 mM potassium phosphate buffer (pH 7.2) containing 18.75% (w/v) dextran 200. The details of the optical cell and spectrophotometer have been reported previously.^52,53^ The distance between the optical cell and the window of the photomultiplier tube was set to <10 mm to minimize the effect of light scattering.^54^ The SRCD spectrum of each sample was measured four times and averaged. The SRCD spectra of solutions containing 10 mM potassium phosphate buffer (pH 7.2) and 18.75% (w/v) dextran 200 were also measured as backgrounds and subtracted from the spectra of the Hsf1 solutions. The helical content of Hsf1 WT and CS5 was analyzed using the BeStSel program.^32^

### Simulations

CGMD simulations were performed with a Martini 3.0 force field^55^ using LAMMPS software.^56^ The molecular structure of Hsf1 was estimated using AlphaFold2.^57^ Among the predicted models, the one having the highest average pLDDT score was selected and used for the simulation. DBD region (residue numbers < 121) was excluded. The crystal structure of BSA (PDB ID:4F5S^58^) was used as the initial configuration in the reference simulation. Atomistic representations of the Hsf1 and BSA models were converted into CG models using the Martinize.py script. Elastic networks^59^ were applied to each monomeric backbone of the BSA protein complex with a distance cutoff of 1.0 nm, using a force constant of 1000 kJ/mol/nm.^56^ We used two different Hsf1 models: Hsf1_flex and Hsf1_helix. While the original conformation of the helices estimated by the AlphaFold2 structure was constrained for the Hsf1_helix model, the helices were unconstrained, and all domains were assumed to be coiled structures for the Hsf1_flex model. Following Periole et al.,^60^ we used harmonic restraints between the backbone beads (BB) separated by four (BB^i^–BB^i+4^) and ten (BB^i^–BB^i+10^) positions, with a force constant of 9250 kJ/mol/nm,^56^ to maintain the secondary structure of α-helices in both the Hsf1_helix and BSA proteins. Eight Hsf1_flex/helix molecules (named chains A, B, C, D, E, F, G, and H for convenience) and six BSA molecules were dispersed in a periodic simulation box and solvated by adding water beads at a protein concentration of 5 wt %. The salt concentration was fixed at 150 mM KCl and the corresponding number of K^+^ and Cl^¬^- charged beads were added. After steepest-descent energy minimization, the systems were equilibrated for 2 ns with a time step of 10 fs at 300 K and 1 atm. Production runs using a 20 fs time step were then performed for 1000 ns in the NPT ensemble at 300 K and 1 atm, and trajectory data were collected every 20 ps. The temperature was maintained using a Nosé–Hoover thermostat^61,62^ and the pressure was controlled using a Parrinello–Rahman barostat.^63,64^ Electrostatic interactions were treated using the damped-shifted force method^65^ with a dampening parameter of 0.2 Å^–1^. A cutoff of 1.1 nm was used for both the LJ and electrostatic interactions. Trajectory data from the last 100 ns were used for the gyration radius, mean square displacement, and distance analysis of the Hsf1 cluster in the equilibrated state. The surface area of proteins was calculated using the surface reconstruction method,^66^ and a spherical probe radius of 0.7 nm VMD^67^ was used to generate the images. To evaluate the reproducibility and consistency of our results, two additional independent runs were conducted for each model using different initial configurations. Analyses from three independent runs are presented as mean and standard deviation values.

For each model, the 2D FES was computed using umbrella sampling simulations.^68^ The free energies were extracted via the weighted histogram analysis method,^69^ as implemented by the Grossfield lab.^70^ The distance between LEU residues and the gyration radius of the droplet were chosen as collective variables. A total of 360 windows were spaced at 0.1 nm intervals, ranging 1.0–3.0 nm of LEU distance and 4.4–8.0 nm of the gyration radius, using a simple harmonic umbrella potential with a force constant of 1500 kJ/(mol nm^2^). The sampling time for each window was 100 ns. From the energy basins identified in the 2D FES, k-means clustering was applied to extract representative snapshots of each metastable state. PCA was performed on the trajectory data within the energy basins to further validate the results obtained from the k-means clustering.

## Supporting Information Available

Confocal immunofluorescence images of cell, differential interference contrast and fluorescence image of droplets, SDS-PAGE gel images, sequence alignment, SEC-MALS profile, circular dichroism spectrum, and coarse-grained molecular dynamics simulation and analysis (PDF)
