# Supplementary material for: Mechanistic Insights Into Oxidative Response of Heat Shock Factor 1 Condensates
Source: JACS Au. 2025 Jan 30;5(2):606–17. doi: 10.1021/jacsau.4c00578 (PMC11863153; doi:10.1021/jacsau.4c00578)
Supplement: Supplementary file 1 — au4c00578_si_001.pdf [file au4c00578_si_001.pdf]

Supporting Information

Mechanistic Insights into Oxidative Response of Heat Shock Factor 1 Condensates

Soichiro Kawagoe[1, ‡], Motonori Matsusaki[1, ‡], Takuya Mabuchi[2,3, ‡], Yuto Ogasawara[4], Kazunori Watanabe[4], Koichiro Ishimori[5], and Tomohide Saio[1,6*]

[1] Institute of Advanced Medical Sciences, Tokushima University, Tokushima 770-8503, Japan

[2] Frontier Research Institute for Interdisciplinary Sciences, Tohoku University, 2-1-1 Katahira, Aoba-ku, Sendai, Miyagi 980-8577, Japan

[3] Institute of Fluid Science, Tohoku University, 2-1-1 Katahira, Aoba-ku, Sendai, Miyagi 980-8577, Japan

[4] Department of Interdisciplinary Science and Engineering in Health Systems, Okayama University, 3-1-1 Tsushimanaka, Okayama, 700-8530, Japan

[5] Department of Chemistry, Faculty of Science, Hokkaido University, Sapporo, Hokkaido 060-0810, Japan

[6] Fujii Memorial Institute of Medical Sciences, Institute of Advanced Medical Sciences, Tokushima University, Tokushima, 770-8503

‡ These authors contributed equally to this work.

[*]Email: saio@tokushima-u.ac.jp

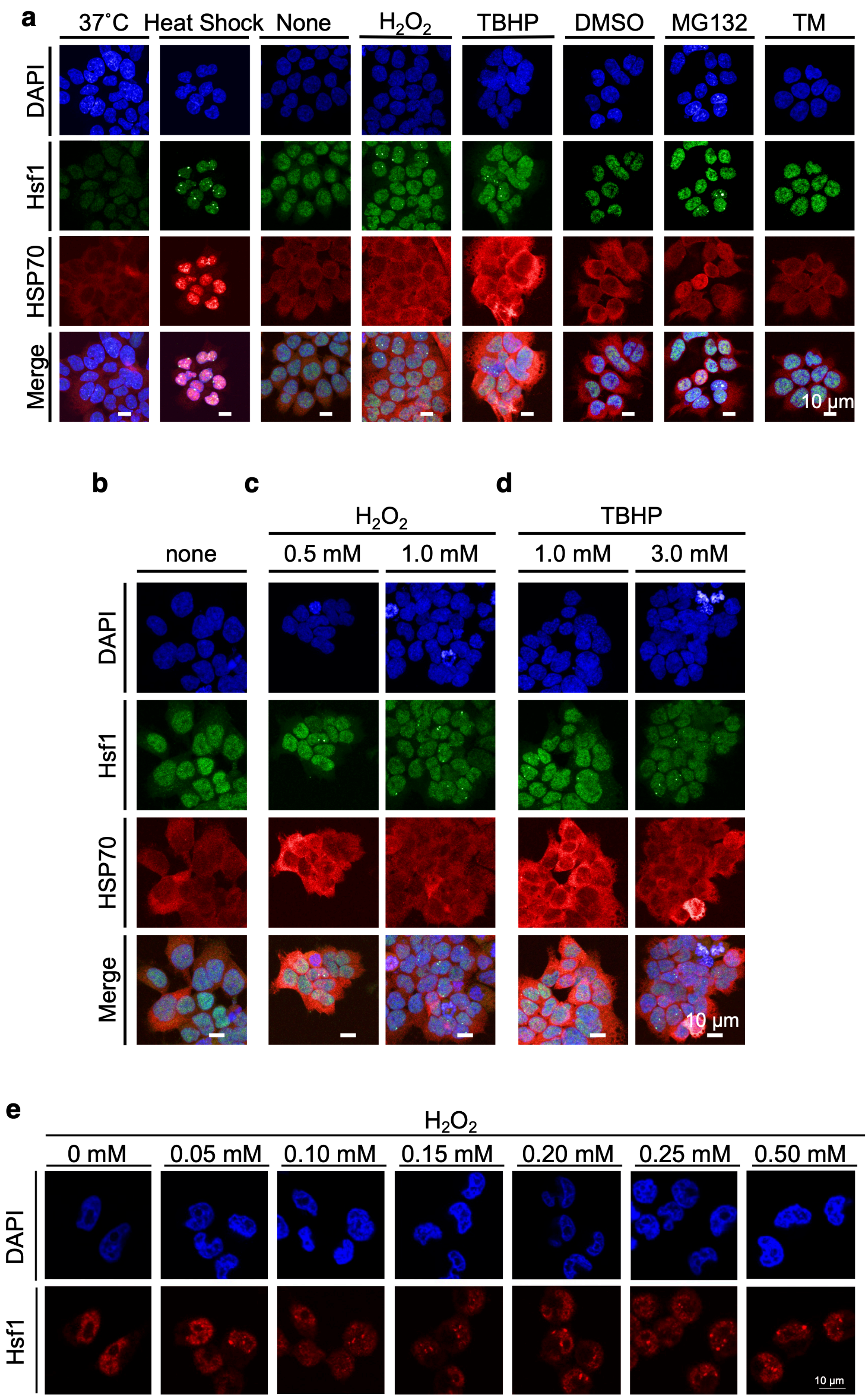
a
37˚C
Heat Shock
None
H2O2
TBHP
DMSO
MG132
TM
DAPI
Hsf1
HSP70
Merge
10 µm
b
c
d
none
H2O2
0.5 mM
1.0 mM
TBHP
1.0 mM
3.0 mM
DAPI
Hsf1
HSP70
Merge
10 µm
e
H2O2
0 mM
0.05 mM
0.10 mM
0.15 mM
0.20 mM
0.25 mM
0.50 mM
DAPI
Hsf1
10 µm

Figure S1. Observation of oxidant dose-dependent nSBs formation. (a) Confocal immunofluorescence images showing subcellular localization and foci formation of Hsf1 in HAP1 cells (scale bar, 10 μm). The cells were then costained with anti-bodies against HSP70 and 4′ ,6-diamidino-2-phenylindole (DAPI). Cells were treated with various stress conditions: 43°C heat shock for 1 h, 1.0 mM H_2O_2 for 1 h, 3.0 mM tert-butyl hydroperoxide (TBHP) for 1 h, and 2 μM MG132 or 2 μg/mL tunicamycin (TM) for 2 h. (b-d) Confocal immunofluorescence images showing the subcellular localization and foci formation of Hsf1 in HAP1 cells (scale bar, 10 μm). Cells were co-stained with an antibody to HSP70 and with DAPI. Cells were treated without agents (b), with oxidative agents: 0.5 mM or 1.0 mM H_2O_2 for 1 h (c) and 1.0 mM or 3.0 mM TBHP for 1 h (d). (e) Confocal immunofluorescence images showing the subcellular localization and foci of Hsf1 in HeLa cells (scale bar 10 μm). Cells were treated with H_2O_2 as oxidative agents at the following concentrations for 2 h at 37˚C: 0 mM, 0.05 mM, 0.1 mM, 0.15 mM, 0.2 mM, 0.25 mM, 0.5 mM. Cells were costained with DAPI.

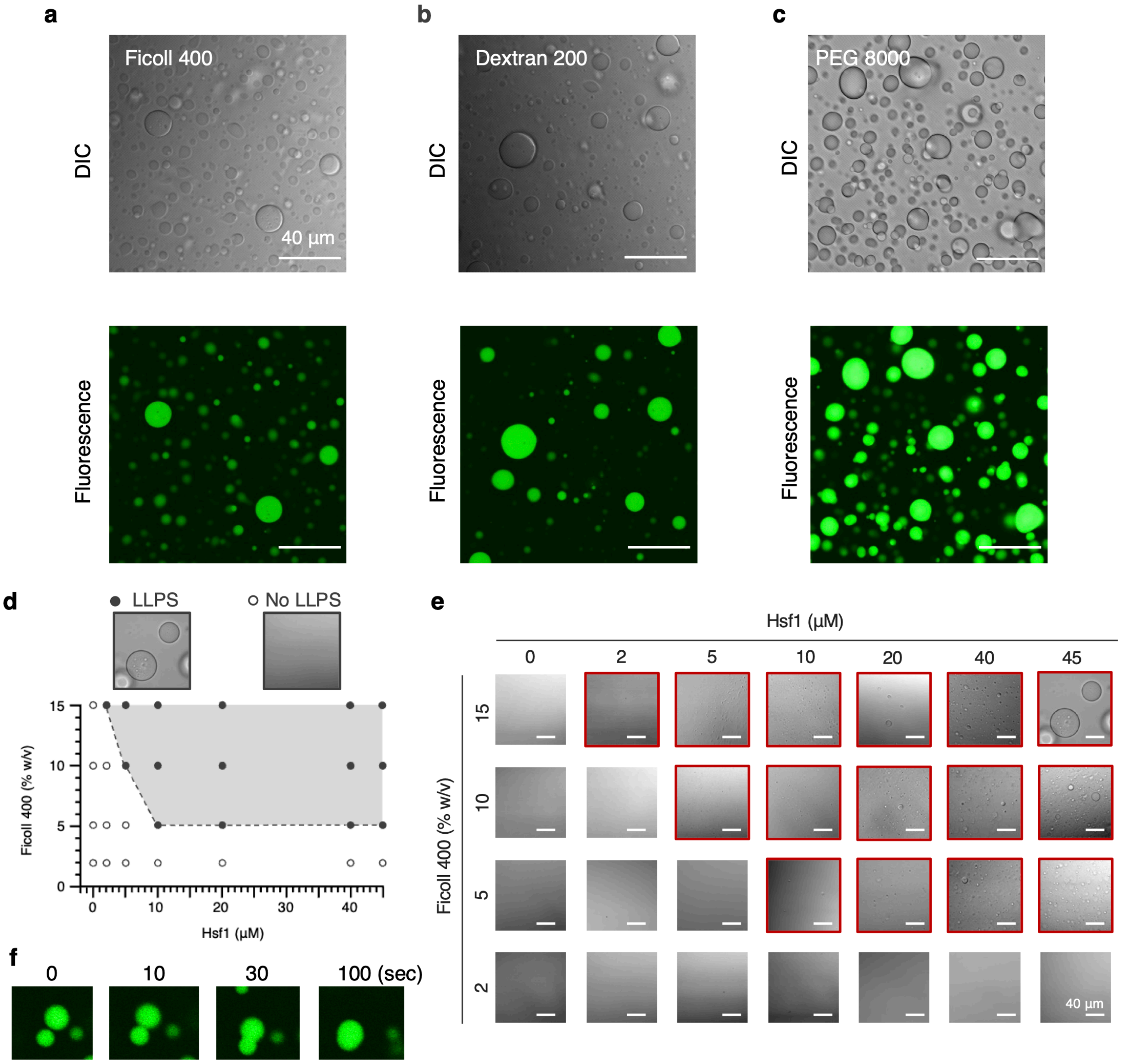

Figure S2. Hsf1 undergoes liquid-liquid phase separation *in vitro*. (a–c) Representative differential interference contrast and fluorescence image showing the droplets formed by Hsf1 mixed with 0.1 eq Hsf1-GFP in the presence of 10 mM DTT and 10 (% w/v) ficoll 400 (a), dextran 200 (b) or PEG 8000 (c). Hsf1 droplet formation was observed in both conditions. Scale bar, 40 µm. (d) Phase diagram of Hsf1 LLPS (Hsf1 concentration versus ficoll 400 concentration). A representative example of LLPS, containing Hsf1 40 µM, ficoll 400 15% (w/v), and a representative example of no LLPS, containing Hsf1 2 µM, ficoll 400 2% (w/v) are presented. (e) Differential interference contrast image showing the droplets used for drawing the phase diagram. Images showing droplet formation are indicated by red box. Scale bar, 40 µm. (f) Time-lapse fluorescence microscopy demonstrating fusion dynamics of Hsf1-GFP droplets.

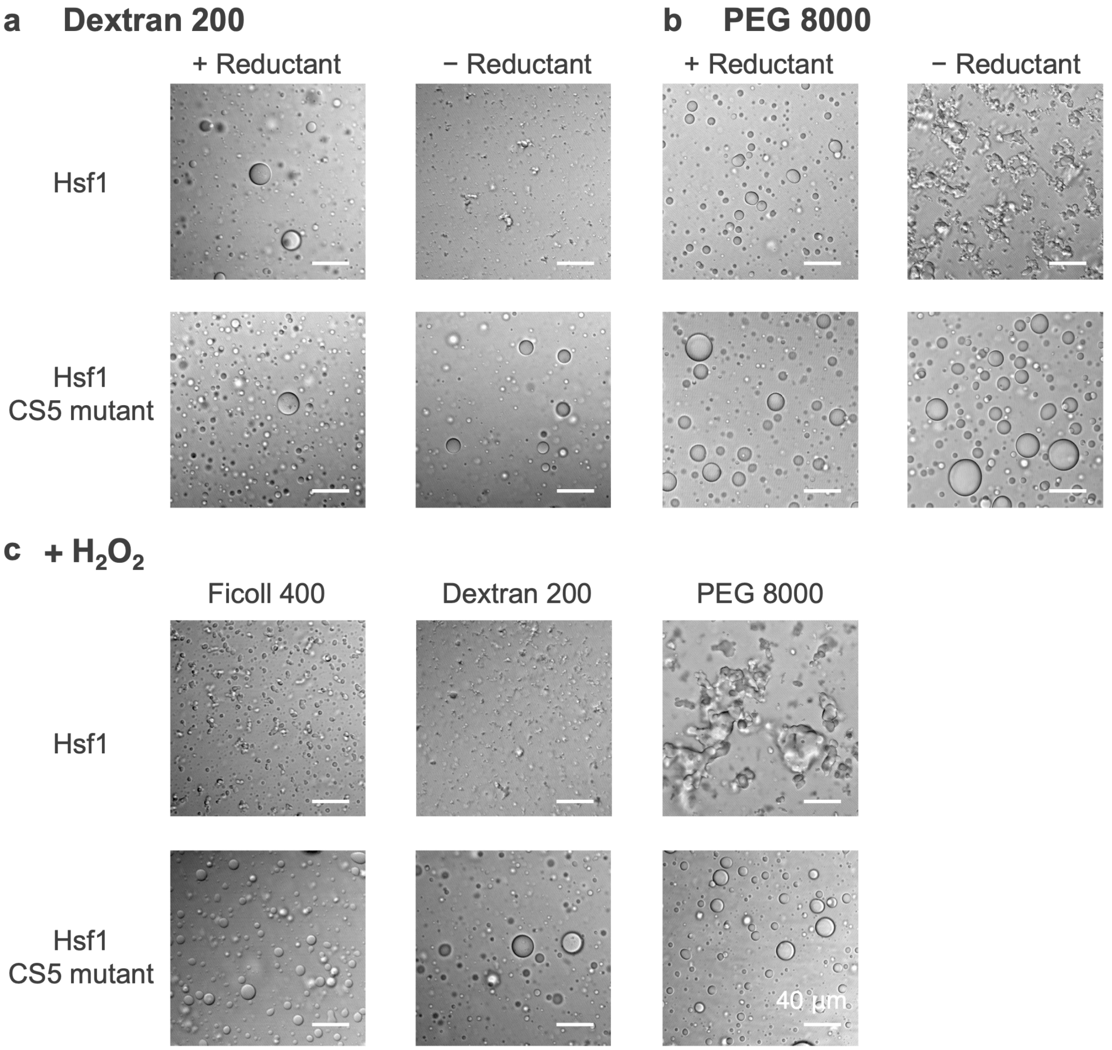

Figure S3. Observation of Hsf1 droplets under various conditions. (a, b) Differential interference contrast images of Hsf1 droplets and Hsf1 CS5 mutant droplets in the presence of 10% (w/v) dextran 200 (a) or PEG 8000 (b). Left and right panels represent the images of Hsf1 in the presence (+ Reductant) and absence (− Reductant) of 10 mM DTT, respectively. Hsf1 droplets have a round globular shape under reductive conditions and a distorted gel-like shape under oxidative conditions. On the other hand, the droplets of Hsf1 CS5 mutant have a round globular shape in all conditions. (c) Differential interference contrast images of Hsf1 droplets and Hsf1 CS5 mutant droplets in the presence of 10 mM H_2O_2. As a crowder, 10% (w/v) ficoll 400 (left panels), 10% (w/v) dextran 200 (center panels), and 10% (w/v) PEG 8000 (right panels) were used for droplet formation. Hsf1 droplets have a distorted gel-like shape, while Hsf1 CS5 mutant droplets have a round globular shape. Scale bar, 40 µm.

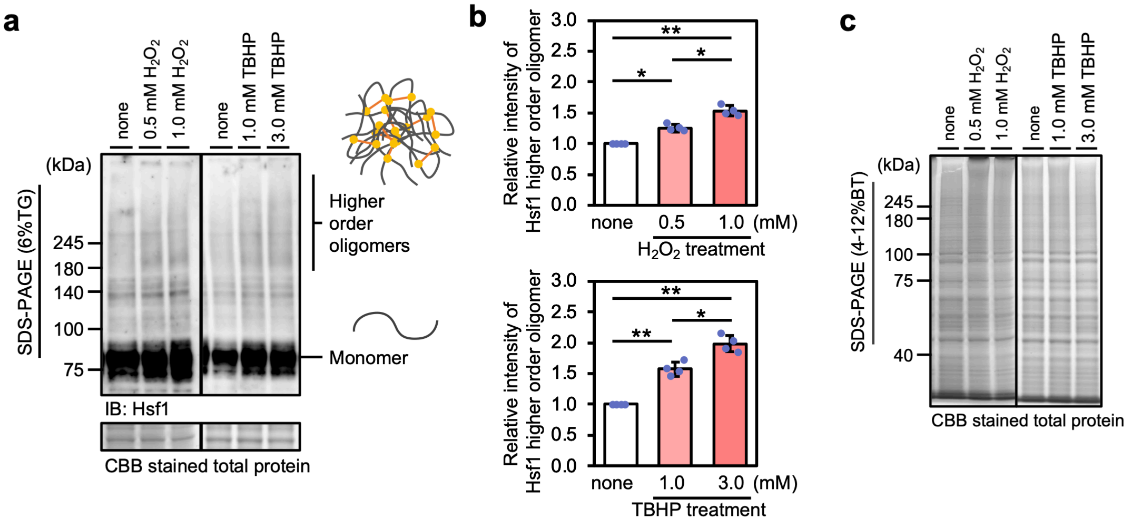

Figure S4. Dose-dependent oligomerization of Hsf1 by oxidants in cells. (a) Oligomeric states of intracellular Hsf1 under oxidative stress. HAP1 cells were treated with an oxidant (H_2O_2 or TBHP). Cell lysates were separated with SDS-PAGE and visualized with anti-Hsf1 antibody. (b) Quantification of the relative signal intensities of the higher-order oligomer forms of Hsf1 compared to the signal intensity of none in (a). Error bars correspond to the means ± s.d. of 3－4 independent experiments. Data were analyzed for statistical significance using the Tukey-Kramer test. *, $p < 0.05$; **, $p < 0.01$. (c) HAP1 cell lysates were loaded in equal proportions to the western blot analysis in (a) and separated by SDS-PAGE. Total protein was visualized by CBB staining.

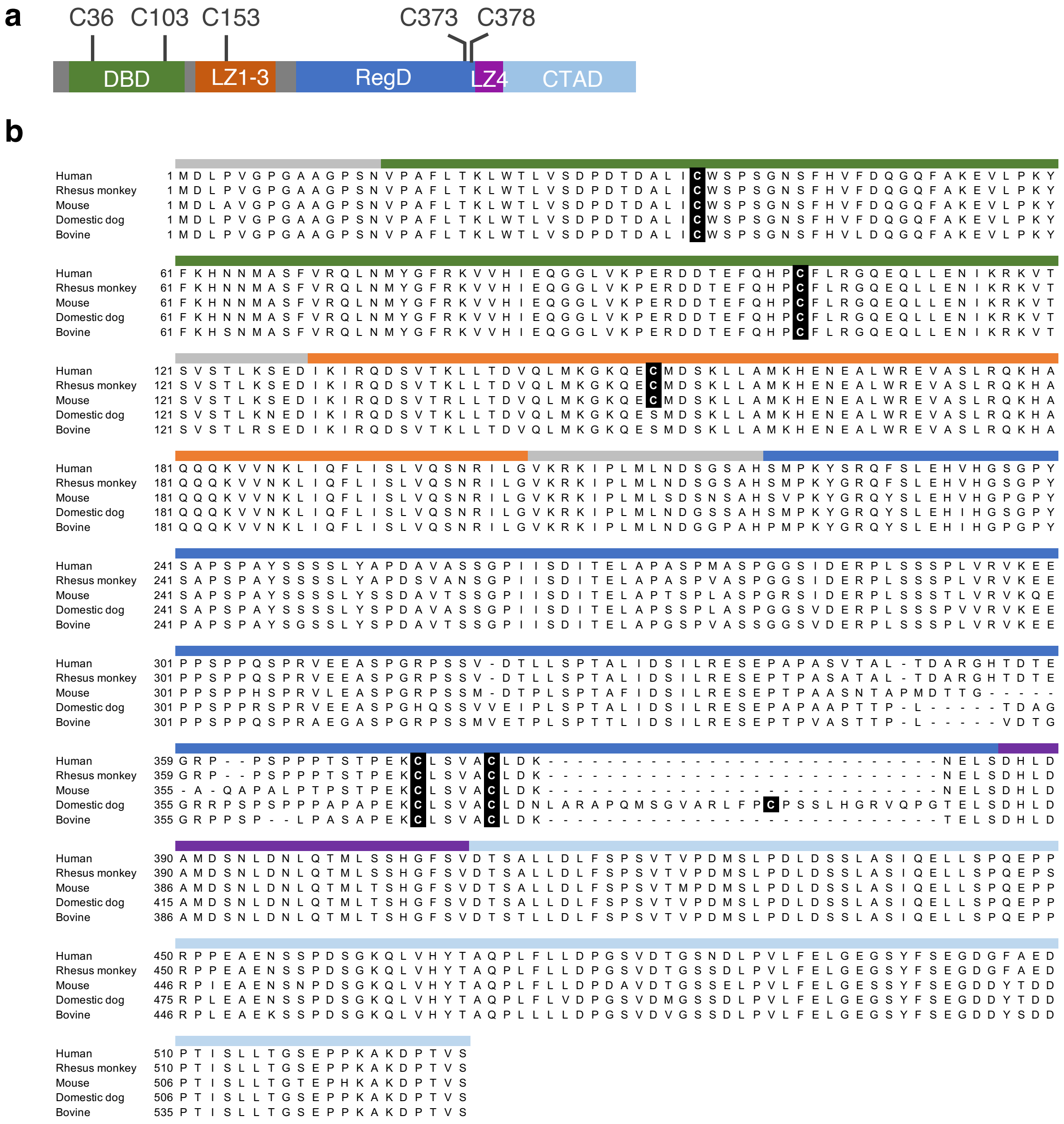

Figure S5. Conservation of the amino acid residues of Hsf1. (a) Domain organization and location of cysteine residues in human Hsf1. (b) Multiple sequence alignment for Hsf1 from a variety of organisms, human (*Homo sapiens*, Q00613), rhesus monkey (*Macaca mulatta*, A0A5K1U5D2), mouse (*Mus musculus*, P38532), domestic dog (*Canis lupus familiaris*, F1PDU7), and bovine (*Bos taurus*, Q08DJ8). Cysteine residues are highlighted in black.

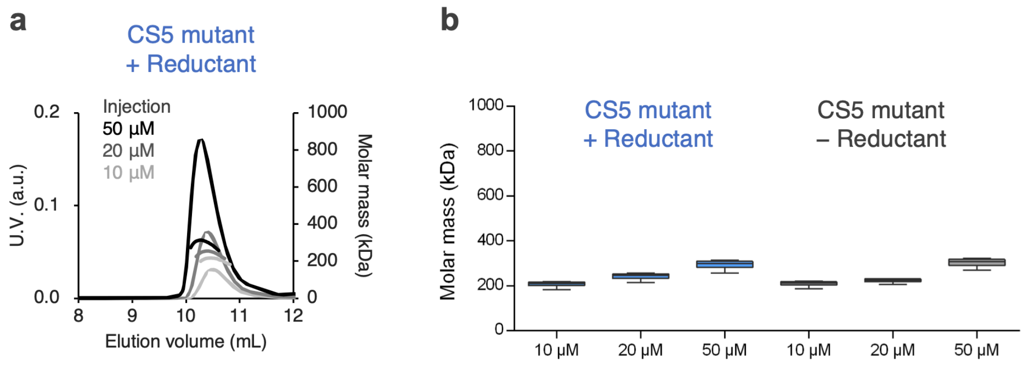

Figure S6. SEC-MALS for Hsf1 CS5 mutant to evaluate the effect of DTT. (a) SEC-MALS results of Hsf1 CS5 mutant injected with 10 mM DTT at varying concentrations. (b) A box and whisker plot of Hsf1 CS5 mutant molar mass in the presence or absence of 10 mM DTT (CS5 mutant +/− Reductant). The addition of DTT to Hsf1 CS5 mutants did not change the molar mass, suggesting that DTT is involved only in the cleavage of disulfide bonds and does not affect the association mode of Hsf1 protomers.

a

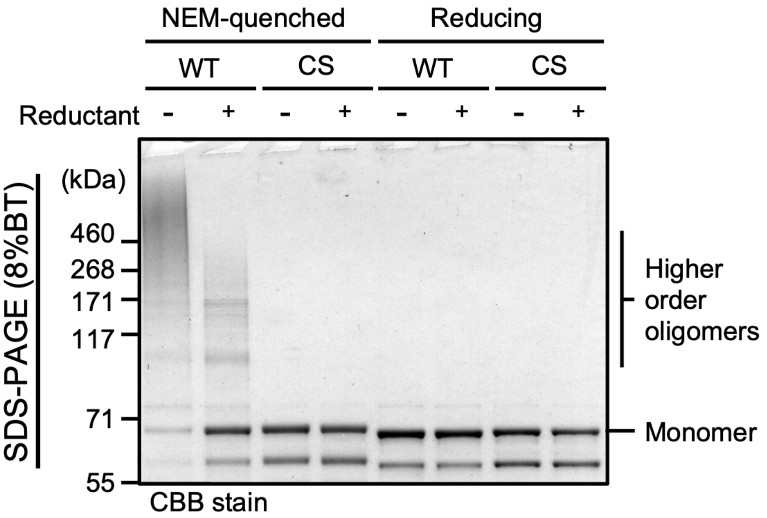

b

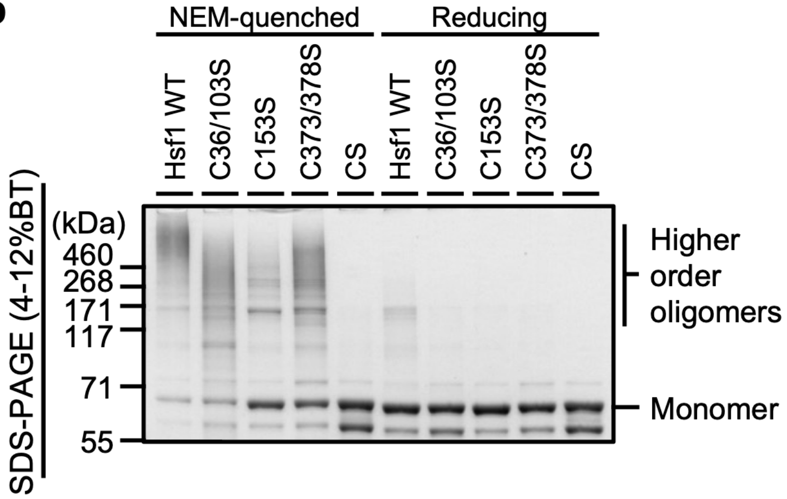

Figure S7. NEM-quenched SDS-PAGE study of the effect of the cysteine mutation on the oligomeric state of Hsf1. (a) Redox states of Hsf1 under reductive conditions. Hsf1 was incubated in the presence or absence of DTT. The samples were quenched with a large excess of NEM (NEM quenched) or sodium dodecyl sulfate (SDS) sample buffer containing a reductant (reducing) and then separated by SDS-PAGE. The reductant (−/+) means the Hsf1 with or without DTT. (b) Oligomeric states of Hsf1 cysteine mutants. Purified recombinant Hsf1 WT and its mutants were treated with NEM or DTT in SDS sample buffer, and samples were separated by SDS-PAGE on a 4–12% gel.

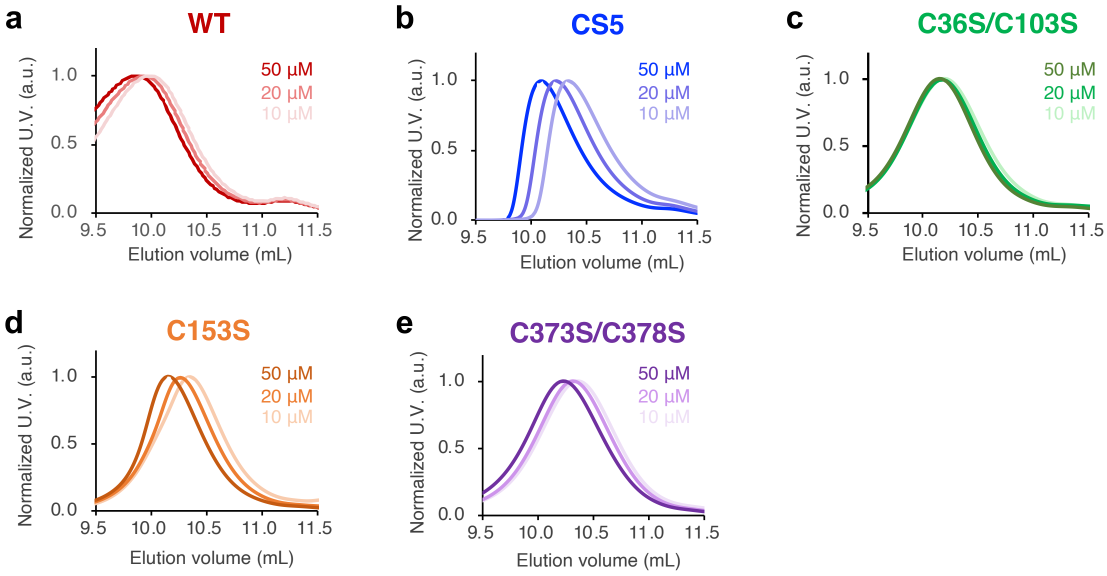

Figure S8. C153 plays critical roles at formation of disulfide-bonded oligomer. (a–e) SEC-MALS of Hsf1 WT (a), Hsf1 CS5 (b), Hsf1 C36S/C103S (c), Hsf1 C153S (d), and Hsf1 C373S/C378S mutant (e) injected at varying concentrations without reductant.

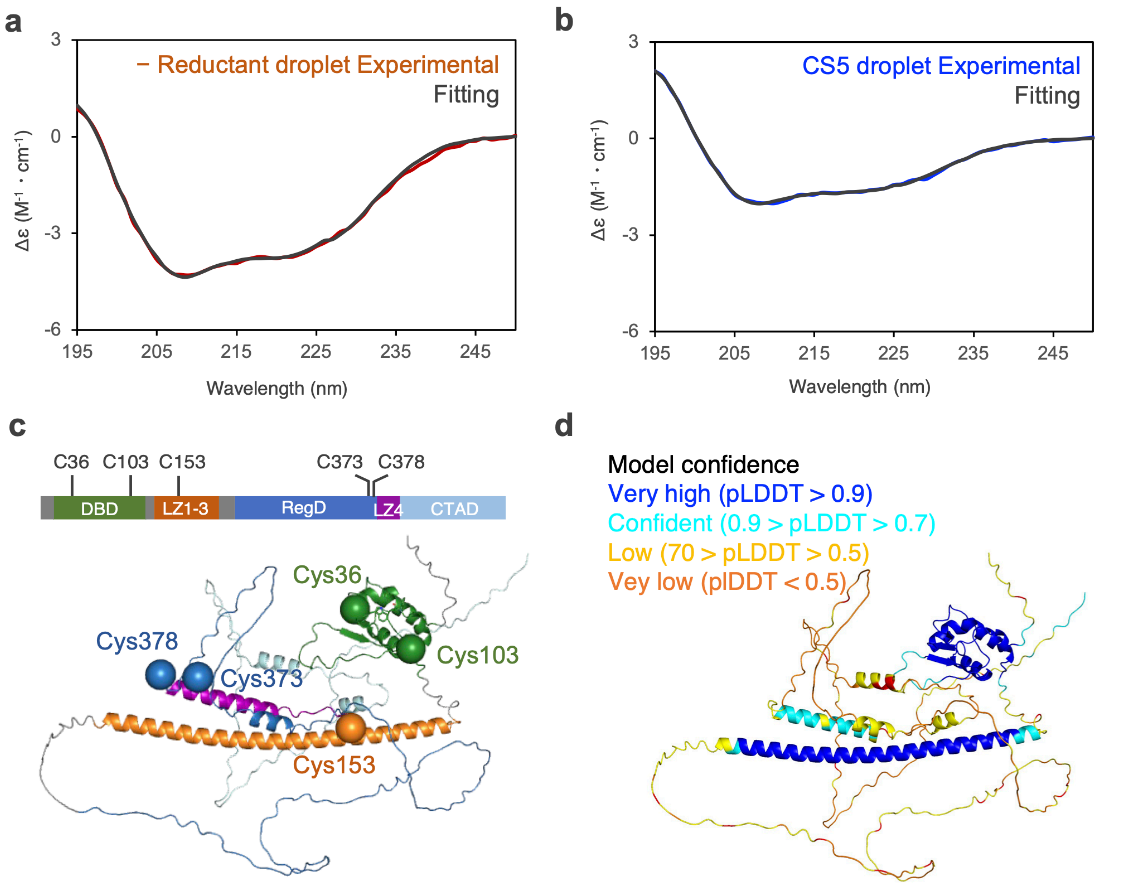

Figure S9. Experimental CD spectra, its curve fitting and predicted structure of Hsf1. (a, b) Fitting of the far CD spectra of the Hsf1 WT (a) and CS5 (b) between 195 and 250 nm at 25°C for determining the secondary structure fractions by the BeStSel program. (c) Domain organization and location of cysteine residues in Hsf1. The configuration of the Hsf1 structure is derived from AlphaFold2. (d) Hsf1 model structures colored by predicted local distance difference test (pLDDT).

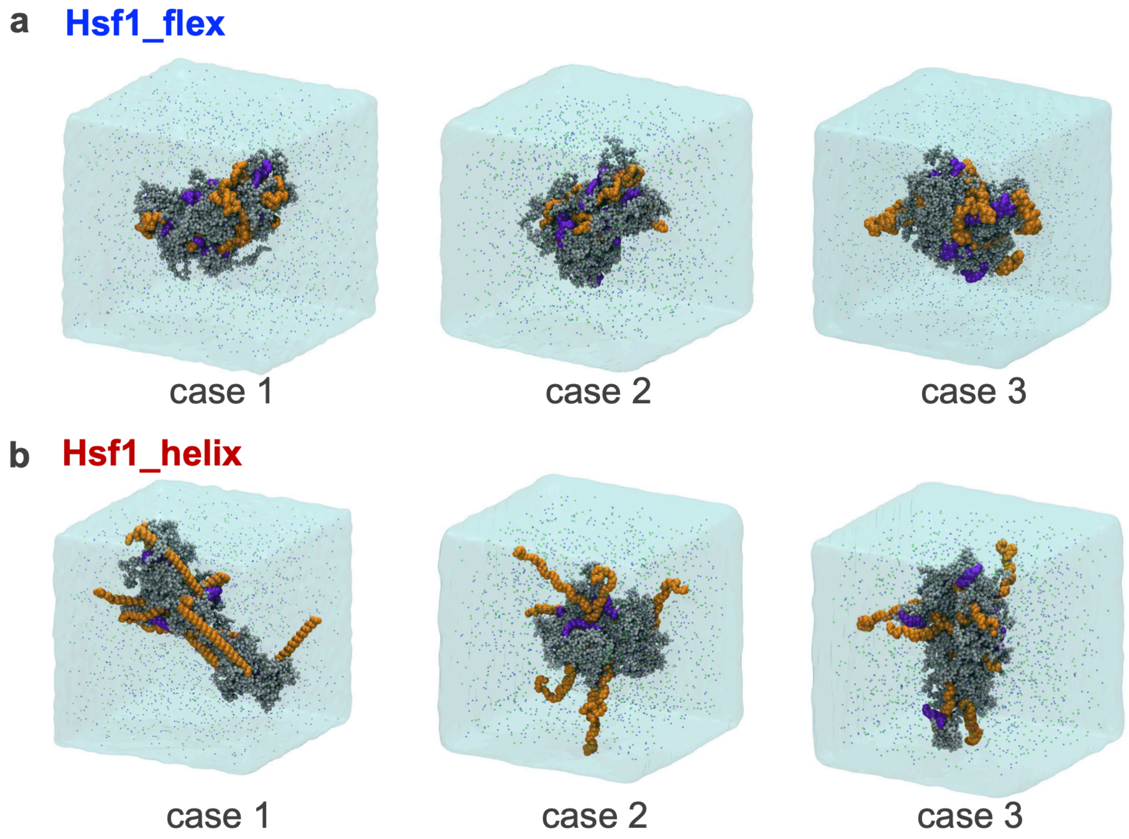

Figure S10. Coarse-grained molecular dynamics simulation on Hsf1. Oligomeric structure of Hsf1_flex (a) and Hsf1_helix (b) in 150 mM KCl aqueous solution after 900 ns simulation. The results of the three independent simulations are shown. K^+ and Cl^- charged beads are shown as small dots in blue and green, respectively, and water beads are shown as surface.

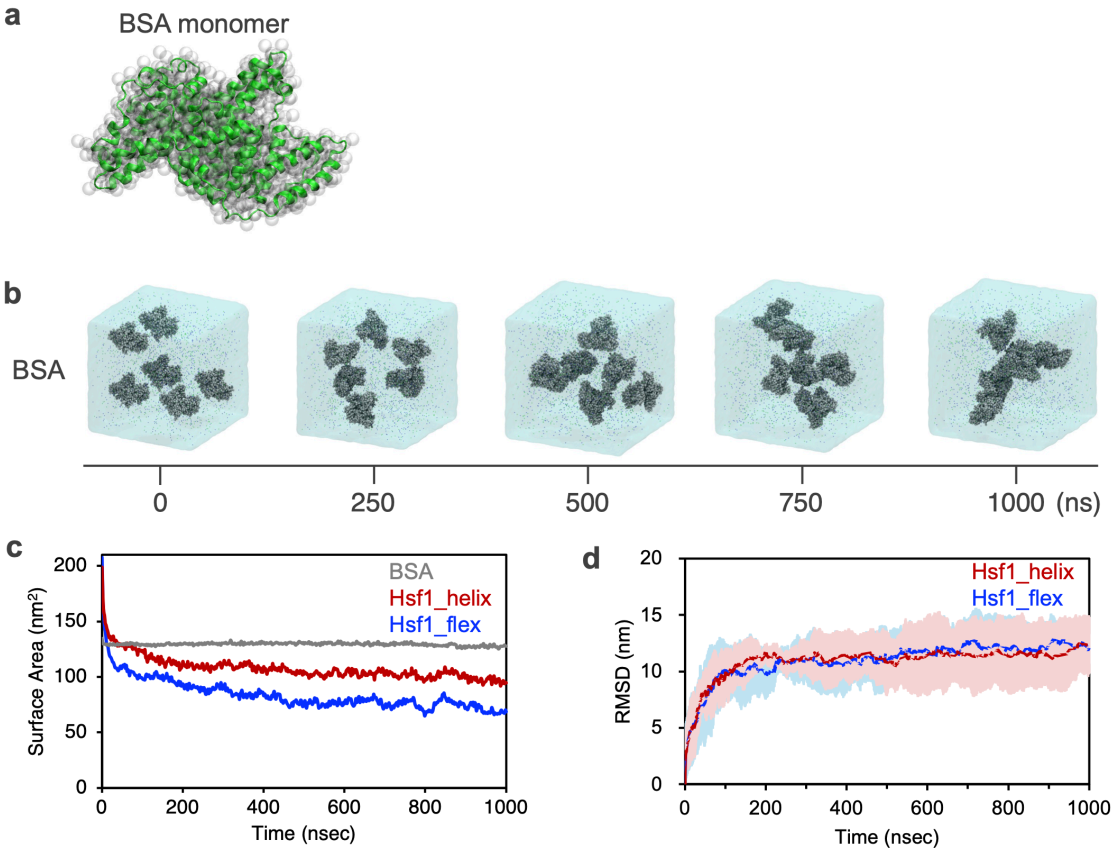

Figure S11. Coarse-grained molecular dynamics simulation on a globular folded-protein BSA as a control. (a) Mapping of the cartoon representation (green) of the BSA structure (PDB ID: 4F5S) to the coarse-grained model. (b) Time evolution of the BSA in 150 mM KCl aqueous solutions. K^+ and Cl^- charged beads are shown as small dots in blue and green, respectively, and water beads are shown as surface. (c) The surface area of BSA proteins as a function of time. A decrease in the surface area of both Hsf1_flex and Hsf1_rigid indicates the assembly of Hsf1. In contrast, a negligible change in the surface area of BSA over time indicates that BSA proteins remain dispersed during the simulation. (d) The root mean square deviation (RMSD) of Hsf1 as a function of time. Error bars correspond to the means ± s.d. of three independent simulations.

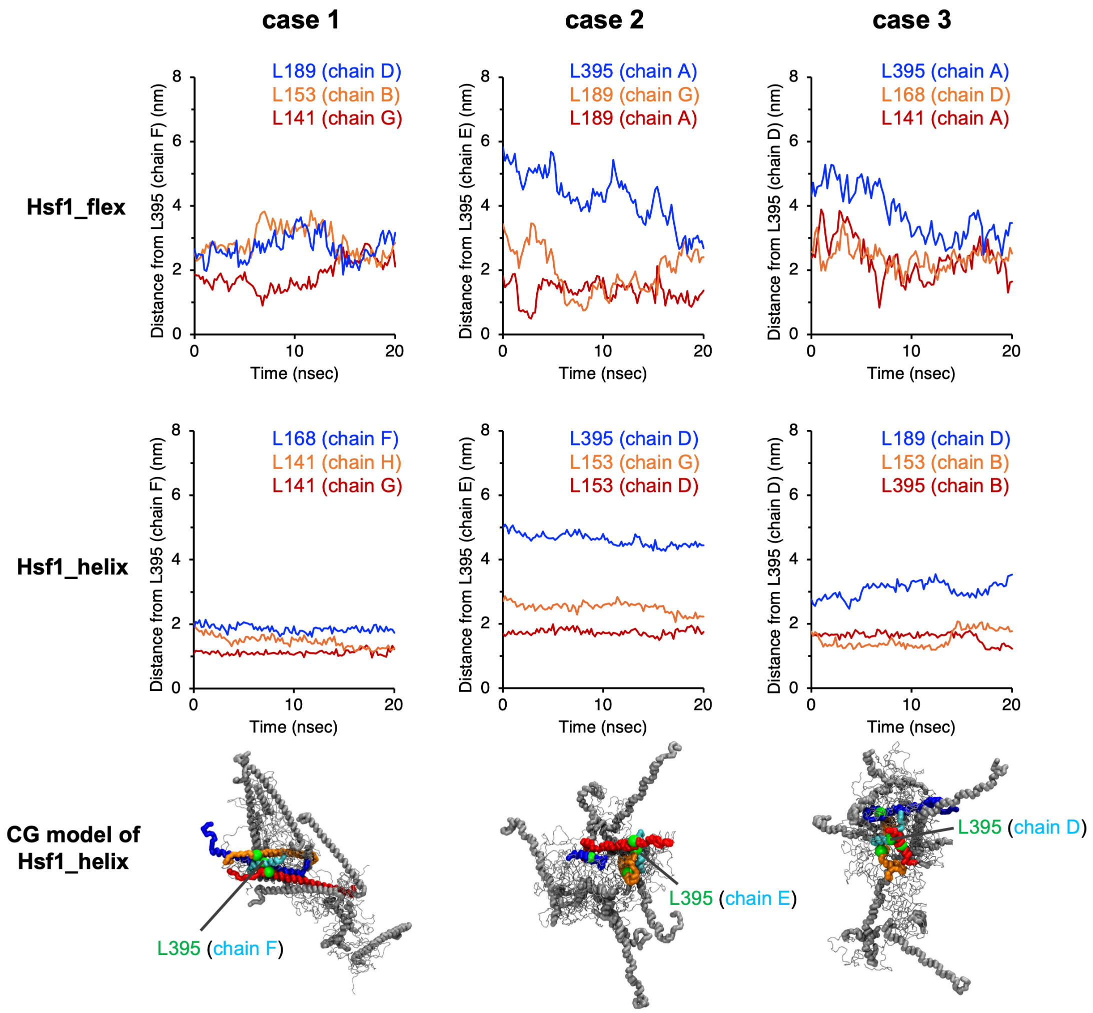

Figure S12. Distance analysis between Leu residues on the helix bundle. (Top and middle panels) Distance between Leu395 (in the LZ4 domain) and the three closest leucine residues in different helices as a function of time. In comparison with Hsf1_flex, Hsf1_helix showed shorter distances and smaller fluctuations over time for all three residues, indicating that the helix bundle structure in Hsf1_helix was stabilized and less mobile. (Bottom panels) Snapshots of the Hsf1 cluster in the Hsf1_helix model at 1000 ns. The LZ helices are shown in a thickened licorice representation, whereas the others are shown in a line representation. A helix bundle structure is shown in a non-gray color: cyan, blue, orange, and red. Leu residues that used to calculate the distance are shown as green spheres.

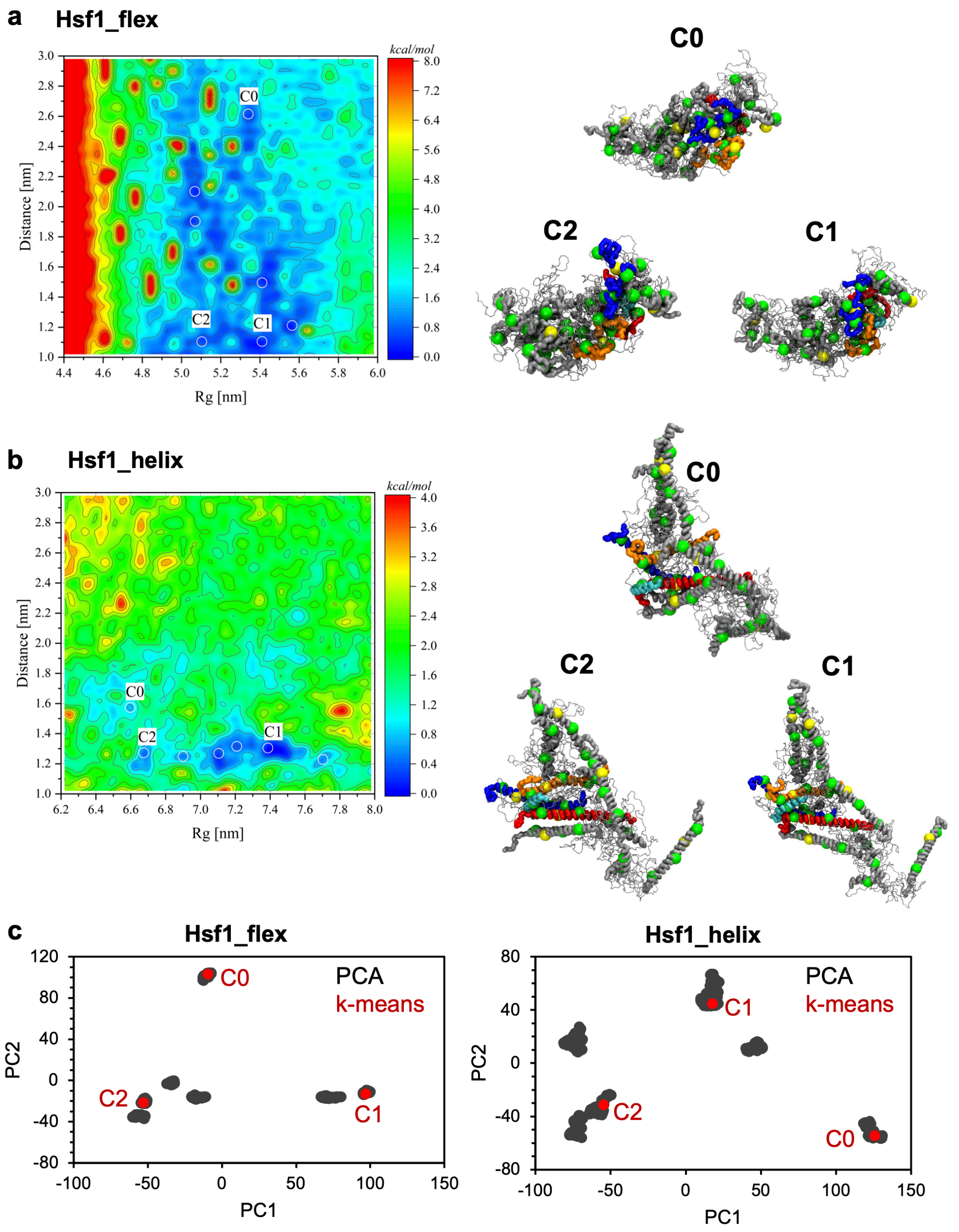

Figure S13. 2D free energy surface using the distance between Leu residues and the gyration radius. (a, b) 2D free energy surface (FES) using two collective variables, specifically the distance between Leu residues and the gyration radius of Hsf1_flex (a) and Hsf1_helix (b). From the energy basins identified in the 2D FES, we applied k-means clustering to extract representative snapshots of each metastable state. (c) Principal component analysis (PCA) to further validate the clustering results obtained through k-means. PCA was performed on the trajectory data from the energy basins, and the first two principal components were used to visualize the main structural variations in the system. The clustering results were then projected onto this reduced space, showing consistent grouping of similar conformations in both the PCA (black dots) and k-means (red dots) results.
